# Aesculetin-mediated recruitment of denitrifying microbiota by resistant rapeseed suppresses *Plasmodiophora brassicae* via nitrate depletion

**DOI:** 10.1093/ismejo/wrag061

**Published:** 2026-05-26

**Authors:** Zhen Zhang, Xingfu Chen, Meihui Wu, Xiuxiu Liu, Lu Chang, Feng Rao, Yuanwei Zhou, Kenichi Tsuda, Chunyu Zhang, Jiasen Cheng, Jiatao Xie, Yang Lin, Yanping Fu, Daohong Jiang, Tao Chen

**Affiliations:** State Key Laboratory of Agricultural Microbiology, Huazhong Agricultural University, Wuhan 430070, China; Hubei Key Laboratory of Plant Pathology, College of Plant Science and Technology, Huazhong Agricultural University, Wuhan 430070, China; State Key Laboratory of Agricultural Microbiology, Huazhong Agricultural University, Wuhan 430070, China; Hubei Key Laboratory of Plant Pathology, College of Plant Science and Technology, Huazhong Agricultural University, Wuhan 430070, China; State Key Laboratory of Agricultural Microbiology, Huazhong Agricultural University, Wuhan 430070, China; Hubei Key Laboratory of Plant Pathology, College of Plant Science and Technology, Huazhong Agricultural University, Wuhan 430070, China; State Key Laboratory of Agricultural Microbiology, Huazhong Agricultural University, Wuhan 430070, China; Hubei Key Laboratory of Plant Pathology, College of Plant Science and Technology, Huazhong Agricultural University, Wuhan 430070, China; State Key Laboratory of Agricultural Microbiology, Huazhong Agricultural University, Wuhan 430070, China; Hubei Key Laboratory of Plant Pathology, College of Plant Science and Technology, Huazhong Agricultural University, Wuhan 430070, China; State Key Laboratory of Agricultural Microbiology, Huazhong Agricultural University, Wuhan 430070, China; Hubei Key Laboratory of Plant Pathology, College of Plant Science and Technology, Huazhong Agricultural University, Wuhan 430070, China; Yichang Academy of Agricultural Science, Yichang 443009, China; State Key Laboratory of Agricultural Microbiology, Huazhong Agricultural University, Wuhan 430070, China; Hubei Key Laboratory of Plant Pathology, College of Plant Science and Technology, Huazhong Agricultural University, Wuhan 430070, China; College of Plant Science and Technology, Huazhong Agricultural University, Wuhan 430070, China; State Key Laboratory of Agricultural Microbiology, Huazhong Agricultural University, Wuhan 430070, China; Hubei Key Laboratory of Plant Pathology, College of Plant Science and Technology, Huazhong Agricultural University, Wuhan 430070, China; State Key Laboratory of Agricultural Microbiology, Huazhong Agricultural University, Wuhan 430070, China; Hubei Key Laboratory of Plant Pathology, College of Plant Science and Technology, Huazhong Agricultural University, Wuhan 430070, China; State Key Laboratory of Agricultural Microbiology, Huazhong Agricultural University, Wuhan 430070, China; Hubei Key Laboratory of Plant Pathology, College of Plant Science and Technology, Huazhong Agricultural University, Wuhan 430070, China; State Key Laboratory of Agricultural Microbiology, Huazhong Agricultural University, Wuhan 430070, China; Hubei Key Laboratory of Plant Pathology, College of Plant Science and Technology, Huazhong Agricultural University, Wuhan 430070, China; State Key Laboratory of Agricultural Microbiology, Huazhong Agricultural University, Wuhan 430070, China; Hubei Key Laboratory of Plant Pathology, College of Plant Science and Technology, Huazhong Agricultural University, Wuhan 430070, China; State Key Laboratory of Agricultural Microbiology, Huazhong Agricultural University, Wuhan 430070, China; Hubei Key Laboratory of Plant Pathology, College of Plant Science and Technology, Huazhong Agricultural University, Wuhan 430070, China

**Keywords:** *Plasmodiophora brassicae*, clubroot, rapeseed, nitrate, root microbiome, denitrifying strains, SynCom, Aesculetin

## Abstract

Clubroot, caused by the protist pathogen *Plasmodiophora brassicae*, is a major threat to cruciferous crop production worldwide. Although plant microbiota is known to influence disease outcomes, the mechanisms underlying microbiota-mediated resistance remain unclear. Here, we investigated the role of plant microbiota in clubroot resistance using two Chinese rapeseed cultivars carrying resistance genes introduced through breeding and their susceptible parental lines. Microbiome profiling revealed that *P. brassicae* infection altered root and rhizosphere bacterial communities, with resistant cultivars displaying distinct assemblages. Functional prediction indicated an enrichment of denitrifying bacteria in the roots of resistant plants following pathogen challenge. Key denitrifying strains were isolated and assembled into a synthetic microbial community (SynCom^18^), which significantly suppressed clubroot development under both controlled and field conditions. In addition to reducing disease severity, microbial treatments improved agronomic traits, including yield and seed quality. Mechanistic analysis revealed a positive correlation between soil nitrate levels and disease severity. Denitrifying strains and SynCom^18^ likely suppressed the development of *P. brassicae* and enhanced plant immunity by reducing soil nitrate levels by ~39.4%. Metabolomic profiling revealed that aesculetin, as a dominant metabolite that is produced by resistance roots and excreted into the rhizosphere to recruit denitrifying bacteria. Our findings show that pathogen-infected clubroot-resistant rapeseed cultivars secrete aesculetin to recruit nitrate-depleting bacteria for resistance against *P. brassicae*. This study elucidates a tripartite microbiota-pathogen-soil nutrient interaction and provides a sustainable biocontrol strategy for cruciferous crops.

## Introduction

Clubroot disease, caused by the soil-borne protist *P. brassicae*, is one of the most damaging diseases of cruciferous crops, with a global distribution reported in over 80 countries [1]. The pathogen infects roots of host plants such as rapeseed (*Brassica napus*), Chinese cabbage, radish, and cabbage [2], inducing wilting, stunting, chlorosis, and, in severe cases, plant death. Yield losses typically range from 20%–30%, but can exceed 60% in heavily infested fields [3, 4]. *P. brassicae* persists in soil as long-lived resting spores that can survive for decades under harsh conditions [4]. These spores spread easily via contaminated soil, water, farm machinery, and infected plant material. Chemical control is largely ineffective against resting spores, and overuse of pesticides raises concerns about environmental impact [5, 6]. Breeding for clubroot resistance (CR) is considered the most cost-effective management strategy. However, effective CR genes are limited to a narrow range of germplasm, primarily derived from *Brassica rapa* fodder turnips [7]. Complicating breeding efforts, *P. brassicae* comprises diverse and evolving physiological pathotypes that often-overcome pathotype-specific resistance [8–10]. Developing resistant cultivars for multiple pathotypes is time-consuming and rarely keeps pace with pathogen evolution. Consequently, clubroot remains a major biotic constraint to sustainable cruciferous crop production. Through molecular marker-assisted selection, the CR locus *PbBa8.1* from turnip (ECD04), which confers resistance to pathotype 4, was introduced into Huashuang 5. This led to the development of Huashuang 5R—the first conventional rapeseed cultivar in China with resistance to clubroot. This variety exhibits resistance to *P. brassicae* pathotypes from regions such as Zhijiang and Huangshan in China [11]. The resistance locus *CRb* from Chinese cabbage CR Shinki into the polima restorer line Bing409 of Huayouza 62 through hybridization and backcrossing, resulting in the development of Huayouza 62R. The developed line showed immune-level resistance to physiological races of *P. brassicae* in the Chinese provinces of Sichuan, Hubei, and Anhui [12]. However, their resistance mechanisms remain unclear.

Nitrogen (N) status directly affects plant defense, modulating both cell structural and signaling components. While moderate N promotes enhances cell wall fortification and metabolic activity that increase pathogen resistance, excessive N can reduce lignin and cellulose deposition, increasing susceptibility to pathogens [13]. N forms also affect immunity: Ammonium (NH₄^+^) uptake and assimilation are critical for rice resistance against sheath blight [14], and NH₄^+^-fed plants exhibit stronger basal responses, activating systemic acquired acclimation and defense against *Pseudomonas syringae* [15]. NH₄^+^ enhances wax deposition, lignification, and systemic resistance in various crops, whereas nitrate (NO₃^−^) modulates SA-, JA-, and ET-dependent defense pathways in a context-dependent manner [16, 17]. Low N levels have been demonstrated to upregulate SA-responsive genes, thereby enhancing disease resistance in tomato. In contrast, high nitrate levels promote susceptibility to pathogens such as *Pseudomonas syringae* and *Oidium lycopersicum* [18]. Similarly, reduced nitrate availability has been found to enhance the tolerance of *Arabidopsis thaliana* to four wild-type *Botrytis cinerea* strains [19]. In addition to plant physiology, N fertilization profoundly shapes rhizosphere microbial communities [20]. Long-term N input can alter microbial diversity and composition, often reducing Acidobacteria [21], whereas enriching Proteobacteria and Actinobacteria [22]. Microbial N-cycling processes—such as symbiotic N fixation, mineralization, nitrification, and denitrification—further impact N availability and plant health [23–26]. Whereas nitrate has been shown to stimulate *P. brassicae* spore germination [27], it remains unclear whether soil nitrate content affects the occurrence of clubroot disease and what the underlying mechanisms may be.

Microbiota are increasingly recognized as integral to plant immunity [28, 29]. Coumarins are a class of naturally occurring phenylpropanoid compounds widely found in plants. In recent years, they have been found to possess significant immunomodulatory and microbiome-regulating functions, playing a key role in plant-microbe interactions. By promoting the colonization of beneficial microorganisms, they help form a microecological environment conducive to plant health [30]. However, whether coumarins play a role in CR has not yet been reported. However, it remains unclear whether the clubroot-resistant varieties Huayouza 62R and Huashuang 5R enhance plant disease resistance by modulating the microbiome through chemical substances. To address this question, we used two clubroot-resistant *B. napus* cultivars, Huayouza 62R and Huashuang 5R alongside their susceptible parental lines [11, 12]. We aimed to: (i) elucidate host-pathogen-microbiota interactions by comparing rhizosphere and root microbiomes; (ii) isolate and evaluate beneficial microbes with biocontrol potential; and (iii) investigate the mechanism underlying microbiota-mediated resistance, with a focus on nitrate depletion as a novel suppressive strategy against *P. brassicae*.

## Materials and methods

### Plant materials, *P. brassicae,* and rapeseed growth conditions

Seeds of the four *B. napus* cultivars—Huayouza 62R (62R), Huayouza 62 (62S), Huashuang 5R (5R), and Huashuang 5 (5S)—were provided by Dr. Chunyu Zhang (Huazhong Agricultural University). Huayouza 62R carries two disease-resistance loci (*PbBa8.1* and *CRb*) and has demonstrated strong field resistance in China [12]. *PbBa8.1* from turnip ECD04 was introgressed into Huashuang 5 via marker-assisted selection to develop Huashuang 5R, which is immune to most Chinese race 4 isolates [11]. *PbBa8.1* with a 2.9 Mb region on chromosome A08. Rapeseed Zhongyou 821 was used for pot experiments, and Zhongshuang 15 for field trials in Zhijiang City. *Arabidopsis thaliana* Col-0 served as the wild-type control. The *f6’h1* mutant (SALK_132418) seeds were provided by Dr. Ke Yu (Henan University). The strain ZJ-1 of *P. brassicae* (race 1, single-spore isolate) was obtained from a diseased rapeseed plant in Zhijiang County, Hubei Province [31]. Plants were grown in a substrate mix of Danish Pindstrup peat: Jiangsu Beilei substrate: vermiculite (8:4:1) under controlled conditions (22°C, 12 h light/12 h dark, 75% RH).

### Seed sterilization and plant cultivation for 16S rRNA sequencing

Seeds of *B. napus* 62R, 62S, 5R, and 5S were surface-sterilized with 50% sodium hypochlorite for 15 min, rinsed thoroughly with sterile distilled water, and sown. For each cultivar, two treatments were established: a non-inoculated control and one inoculated with *P. brassicae* (inoculated 9 days after sowing with 1 × 10^7^ spores/ml suspension). No pesticides were applied during cultivation to avoid microbiome disturbance. After 38 days, root and rhizosphere soil samples were collected (eight replicates per treatment), flash-frozen in liquid nitrogen and stored at −80°C.

### DNA extraction and amplicon sequencing data analysis

Total DNA was extracted using the DNeasy PowerSoil Kit and quantified with a NanoDrop 2000. The V5–V7 region of bacterial 16S rRNA gene was amplified with primers 799F/1193R and sequenced on the NovaSeq 6000 System (Illumina; Personalbio, Shanghai) [32]. Raw paired-end FASTQ files were quality-filtered using fastp (v0.20.1) [33], and denoised to amplicon sequence variants (ASVs) with the DADA2 plugin in QIIME 2 [34, 35]. Taxonomy was assigned using the Silva 138.2 database with a naive Bayes classifier [36, 37]. Beta diversity was assessed by principal coordinate analysis (PCoA) based on Bray–Curtis dissimilarity using R package vegan (https://www.R-project.org/; see Table S1 for R code). Sequencing depth was evaluated using rarefaction curves of observed ASVs.

### Construction and analysis of microbial co-occurrence networks and prediction of microbiota functions

Co-occurrence networks of the root microbiome in resistant and susceptible cultivars were analyzed. Samples were divided into four groups based on *P. brassicae* inoculation status (inoculated vs. non-inoculated) and sampling compartment (root vs. rhizosphere). The top 80 most abundant amplicon sequence variants ASVs from the feature table were selected, and their correlation matrices were computed using R. The co-occurrence networks were then visualized using Gephi (v0.8). Functional annotation of the root microbiome in *P. brassicae*-inoculated samples (resistant vs. susceptible cultivars) was performed using the FAPROTAX (v1.2.5) analysis database [38]. Differential metabolic and ecological functions between resistant and susceptible cultivars were then analyzed and visualized using STAMP.

### Pathogen inoculation assays and clubroot disease assessment

*P. brassicae* inoculation followed established protocols [39]. Resting spores were extracted from surface-sterilized root galls, purified via sucrose density centrifugation, and sterilized using 2% chloramine-T and antibiotics. Ten-day-old rapeseed seedlings were inoculated with 1 ml of spore suspension (1 × 10^7^ spores/ml) [39]. Clubroot severity was assessed 4–5 weeks post-inoculation (pot) or over two months (field) using a 0–5 scale [40].

### Screening of strains inhibiting *P. brassicae* resting spore germination

Bacterial strains were screened for inhibition of *P. brassicae* resting spore germination based on previously reported with modifications [41]. Rapeseed root exudates (RE) were collected by surface-sterilizing seeds with 3% NaClO, rinsing, incubating in sterile water at 22°C for 5–10 days, and filtering the liquid through a 0.22 μm membrane. Bacterial fermentation broth (BFB) was prepared by culturing strains in R2A medium at 28°C with shaking for 48 h, followed by centrifugation and filtration. For the assay, 1 ml of resting spore suspension was centrifuged, mixed with 500 μl BFB and 500 μl RE, incubated in the dark at 22°C for 72 h, stained with DAPI (4′,6-diamidino-2-phenylindole), and observed under fluorescence microscopy. DAPI staining visualizes nuclei: ungerminated resting spores exhibit fluorescence, whereas fluorescence disappears after germination into primary zoospores. The experiments were repeated at twice with 5–8 biological replicates per experiment, with similar results.

### Giltay medium

Solution A: KNO₃ 1 g, asparagine 1 g, 1% bromothymol blue (BTB) in ethanol 5 ml, distilled water 500 ml. Solution B: Sodium citrate 8.5 g, MgSO₄·7H₂O 1 g, FeCl₃·6H₂O 0.05 g, KH₂PO₄ 1 g, CaCl₂·2H₂O 0.2 g, distilled water 500 ml. Mix Solutions A and B, adjust pH to 7.0, sterilize, and solidify as agar plates. Inoculate with fresh bacterial colonies and incubated at 28°C for 2–3 days. A color change to blue indicates preliminarily denitrification potential.

### Pot experiment

Seeds were surface-sterilized with 3% NaClO for 10 minutes, rinsed thoroughly, and then sown (32 plants per treatment). On day 9, treated plants received 10 ml of bacterial suspension (3 × 10^9^ CFU/ml, OD_6_₀₀ = 1.5) per plant, whereas control plants received sterile water. The following day (day 10), all plants were challenge-inoculated with 1 ml of *P. brassicae* resting spore suspension (1 × 10^7^ spores/ml). Additional bacterial inoculations were given on days 7, 14, and 21 post-pathogen inoculation. Plants were harvested 28 days for pathogen challenge for disease assessment and qPCR-based pathogen biomass quantification. The experiment was repeated twice with similar results.

### Gnotobiotic plant growth system

Nine plastic pots were arranged per sterile container: one central pot with water-absorbent silica gel, eight for rapeseed. A 2:1 (v/v) mixture of autoclaved clay (700 ml) and LS nutrient solution (350 ml) was added to cultivation pots. Containers were covered with sterilized lids, double-bagged, and autoclaved twice (121°C, 30 min) on consecutive days. Surface-disinfected seeds were transferred (3–4 seeds/pot) using sterile forceps. Seedlings were thinned to one plant/pot on day 5. Bacterial challenge (5 ml suspension, 3 × 10^9^ CFU/ml, OD_6_₀₀ = 1.5) was applied on day 9. Pathogen inoculation (1 ml of filtered *P. brassicae* resting spores, 1 × 10^7^ spores/ml) followed on day 10 via root drenching. Weekly bacterial booster inoculations were given. Disease severity was assessed 28 days post-pathogen inoculation; roots were collected for qPCR-based pathogen quantification. Experiments were repeated at twice with 16 replicates each, with similar results.

### Field experiments

Field biocontrol trials were conducted in Zhijiang City, Hubei Province, from September 2022 to May 2024. The experiment used randomized plots design, with each plot measuring 5 m × 2 m and containing seven rows of rapeseed (Zhongshuang 15). Each row, planted along the length, contained 20 plants. The two outermost rows served as protective borders. Each treatment had six replicate plots: three for disease index assessment and three for yield measurement. Seeds were surface-sterilized, soaked in a bacterial suspension (6 × 10^8^ CFU/ml, OD_6_₀₀ = 0.3) for 5 h at 28°C, air-dried overnight, and sown. At sowing, each plot received 2.5 L of bacterial suspension (4 × 10^9^ CFU/ml), the chemical control treatment received cyazofamid (1:2000 dilution). Additional bacterial applications were made at 14, 21, and 28 days post-sowing. Clubroot severity was assessed at the seedling stage (76 or 63 day after sowing). Approximately 50 plants per plot were randomly sampled. The Disease Index (DI) was calculated as: DI = [Σ(Grade × Number of plants in grade) × 100] / (4 × Total plants). Disease grades were 0–4. Agronomic traits were assessed at seedling (100 days after sowing), flowering (176 or 181 days after sowing), and maturity (> 200 days after sowing) stages. At maturity, each plot was harvested separately. Seeds were sun-dried, threshed, and analyzed for yield, thousand-seed weight, and oil content (determined by a near-infrared spectrometer).

### Measurement of relative biomass of *P. brassicae*

Total DNA were extracted from roots using the CTAB method [42]. DNA was diluted to 100–200 ng/μl and used as a template for qPCR with primers for *Pbactin* (AY452179.1) and *BnaGDI1*. The reaction mixture contained 5 μl 2X supermix, 0.5 μl each primer, 2 μl DNA, and 2 μl ddH₂O. Cycling conditions: 95°C for 10 min, 45 cycles of 95°C for 15 sec and 57°C for 15 sec, melt curve 65°C to 95°C (0.5°C/5 s). Reaction was run on a Bio-Rad CFX 96. Relative *P. brassicae* biomass was calculated via 2^−ΔΔCt^ method using Ct values of *Pbactin* and *BnaGDI1*. Data are from two independent experiments, each with 3–4 biological replicates and 3 technical replicates. Primer sequences are in Table S2.

### ROS quantification

ROS was measured as described [43]. Briefly, seven-day-old rapeseed seedlings were pre-cultured for 3 days in Hoagland solutions under three nitrogen levels: LN (6 mM NO₃^−^-N), HN (30 mM NO₃^−^-N), or DHN (60 mM NO₃^−^-N). Root segments (0.5 cm) were excised and incubated overnight in corresponding nutrient solutions in a 96-well microplate. After replacing the medium with reaction buffer (20 μg/ml HRP, 15 μM L-012, 50 μg/ml chitin), luminescence was immediately recorded using a plate reader.

### RNA extraction and qPCR analysis

RNA extraction and qPCR analysis were described [39]. Total RNA was extracted using TRIpure reagent (Aidlab, RN0102, Beijing). Approximately 2–5 μg of RNA was reverse-transcribed into cDNA with the One-Step gDNA Removal and cDNA Synthesis Kit (TransGen, AT311–02, Beijing). Transcript levels of *PR1*, *PR2*, and *PDF1.2* were analyzed by qPCR and normalized to rapeseed *actin* as the internal control. Each treatment included 2–3 technical replicates and 3 biological replicates, and the experiment was repeated twice independently. Primer sequences are listed in the Table S2.

### Analysis of total nitrogen, nitrate, and ammonium content

Five grams of fresh soil were weighed into a 50 ml centrifuge tube, mixed with 25 ml of KCl solution, and shaken for 1 h. After settling for 30 min, the supernatant was filtered. Then, 4 ml of the filtrate was collected, mixed with2 ml of 1 mol/L HCl, and diluted to 8 ml with KCl. The solution was stored at 4°C. Standard solutions for NH₄^+^-N NO₃^—^N (0–50 mg/L) were prepared. Reagents (CuSO₄, ZnSO₄, colorimetric reagent, hydrazine sulfate, and sodium salicylate) were prepared per flow injection analyzer manual. NH₄^+^-N and NO₃^—^N contents were measured as described [44]. The experiment was repeated twice with similar results.

### Non-targeted metabolomics

Root exudate was collected from rapeseed seedlings 30 days after inoculation with *P. brassicae*. Rhizosphere soil tightly bound to the roots was flash-frozen in liquid nitrogen, lyophilized, and resuspended in 100 μl deionized water. Metabolite profiling was performed by LC–MS/MS (Thermo UHPLC-Q Exactive) equipped with an ACQUITY BEH C18 column (100 mm × 2.1 mm i.d., 1.7 μm; Waters) [45]. Quality control samples with a relative standard deviation (RSD) > 30% were excluded, and data were log10-transformed. Orthogonal partial least squares-discriminant analysis (OPLS-DA) was performed using the R package ropls (v1.6.2), with model stability assessed via 7-fold cross-validation. Metabolites with a variable importance in projection (VIP) score > 1 and *P* < .05 (Student’s *t* -test) were considered significant. Metabolic pathway analysis was conducted using MetaboAnalyst.

### Chemotaxis assay

A modified capillary assay was conducted following established protocols [46]. Briefly, a 25-gauge needle (2 cm) served as the chemotaxis capillary, connected to a 1 ml syringe containing 200 μl of phenylpropanoid-related metabolites. The needle tip was inserted into a 200 μl pipette tip containing 100 μl of bacterial suspension (OD_600_ = 0.1 in PBS). After 2 h incubation at room temperature, the capillary contents were serially diluted and plated on LB agar (1.5%). Bacterial accumulation was quantified as mean colony-forming units (CFUs) from triplicate plates. The final accumulation value per treatment was derived from three independent capillary assays. The relative chemotaxis index (RCI) was calculated as: RCI = Accumulation (test capillary)/Accumulation (negative control capillary). An RCI ≥ 2 was considered indicative of significant chemotactic attraction. Experiments were repeated at twice with 4 biological replicates each, with similar results.

### Measurement of biofilm formation

Biofilm formation was assessed as described [47]. Briefly, bacterial suspension (OD_600_ = 0.02) in lysogeny broth medium were added to 24-well plates containing phenylpropanoid metabolites (or no metabolites as control). After 12–24 h at 28°C, wells were washed, stained with 0.1% crystal violet for 30 min, and destained with ethanol: acetic acid solution (4:1, v/v). Absorbance was measured at 530 nm. Experiments were repeated at twice with three biological replicates each.

### Gnotobiotic bacterial quantification assay

Bacteria quantification in the Arabidopsis rhizosphere and roots was performed as described [48], with minor modifications. Briefly, seeds (Col-0 and *f6’h1*) were germinated on Teflon mesh discs placed in 24-well plates containing 500 μl of half-strength MS medium supplemented with 1% sucrose. After 10 days, the medium was replaced with 450 μl of sucrose-free medium (half-strength MS with 0.1% MES buffer) to ensure bacterial dependence on plant root exudates as the sole carbon source. Two days later, 50 μl of bacterial suspension (OD_600_ = 0.0002) was inoculated per well. Aesculetin or DMSO was added to designated wells. After an additional 2 days, samples from both the liquid medium and the roots were collected for bacterial quantification. Plants with translucent or water-soaked leaves were discarded. Bacterial load in the liquid phase was determined by dilution plating planting, representing rhizosphere colonization levels.

### High-performance liquid chromatography analysis of aesculetin

To determine whether *P. brassicae* induces differential production of aesculetin in resistant and susceptible rapeseed cultivars during early infection, 10-day-old seedlings were transplanted into 2 ml tubes and inoculated with 1 ml of resting spore suspension (1 × 10^7^ spores/ml). The inoculated plants were incubated in a growth chamber for 24 hours, after which aesculetin content in both roots and spore-free exudates was analyzed using high-performance liquid chromatography (HPLC). Control plants were treated with sterile water instead of the resting spore suspension.

### Statistical analyses

Data were processed with Microsoft Office, and figures were prepared using GraphPad Prism 8.0. Statistical significance was assessed with Student’s *t*-test, or one-way ANOVA followed by Duncan’s multiple range test (SPSS Statistics 26.0). A *P* value <0.05 was considered statistically significant.

## Results

### Clubroot pathogen reshapes root and rhizosphere microbiomes

**Figure 1:**
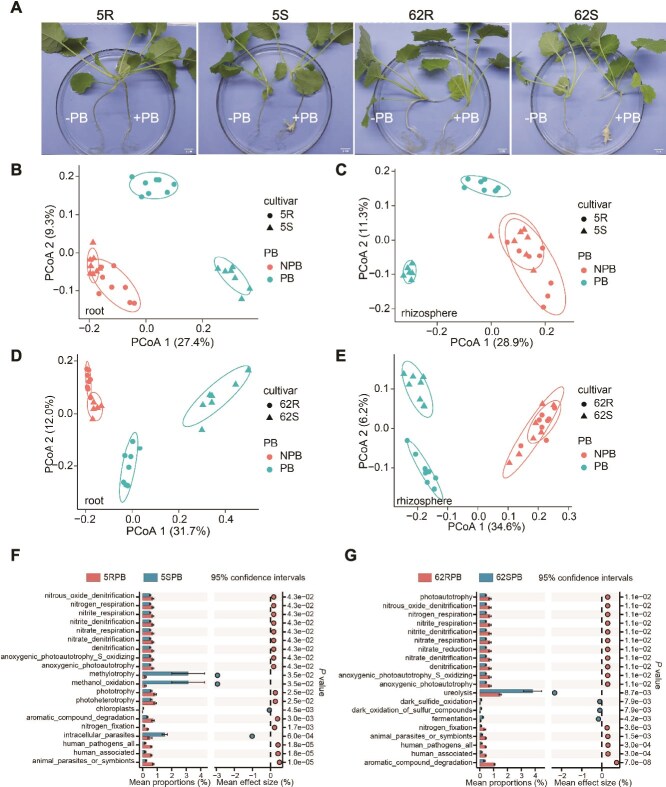
Root and rhizosphere microbiota composition in clubroot-resistant and susceptible cultivars following *P. brassicae* inoculation. (A) Phenotypic comparison of resistant cultivars (Huashuang 5R [5R] and Huayouza 62R [62R]) and susceptible cultivars (Huashuang 5 [5S] and Huayouza 62 [62S]) at 29 days post-inoculation with or without *P. brassicae* (scale bar = 2 cm). (B-E) Principal coordinate analysis (PCoA) of Bray–Curtis distances showing: (B, D) root microbiota and (C, E) rhizosphere microbiota composition in resistant versus susceptible cultivars following *P. brassicae* treatment. Color indicates inoculation status (with/without *P. brassicae*), whereas shape represents different cultivars (PERMANOVA, *P* < .05 by Adonis test). (F, G) Functional annotation of root bacterial communities using FAPROTAX analysis reveals metabolic differences between resistant cultivars (5R, 62R) and susceptible cultivars (5S, 62S). Extended error bar visualization of significantly different metabolic pathways (FDR < 0.05). Panel organization: (F) 5R vs. 5S comparison, (G) 62R vs. 62S comparison.

To investigate the relationship between microbiota and CR, we performed 16S rRNA gene amplicon sequencing on the two resistant cultivars 5R and 62R and their susceptible parental lines 5S and 62S under greenhouse conditions. Each cultivar was grown with or without *P. brassicae* inoculation. At 29 days post-inoculation (dpi), aboveground growth remained healthy (Fig. S1A), but root galls were only observed in susceptible lines (Fig. 1A). Rarefaction curve analysis showed that the number of observed species plateaued with increasing sequencing depth, confirming adequate sampling coverage (Fig. S1B, C). PCoA revealed overlapping microbiome compositions between 5S and 5R under control conditions, but clear separation after pathogen inoculation; similar patterns were observed between 62S and 62R (Fig. 1B, E). This indicates that *P. brassicae* infection alters bacterial compositions in roots and rhizospheres, and that resistant and susceptible genotypes respond differently to the infection.

Pathogen challenge selectively enriched Actinobacteria and γ-Proteobacteria in resistant roots, taxa associated with disease suppression (Fig. S2). Co-occurrence network analysis showed that resistant cultivars maintained more complex and stable microbial networks than susceptible lines (Fig. S3). Functional prediction using FAPROTAX analysis revealed significant enrichment of N cycling functions—N fixation, nitrate/nitrite reduction, and denitrification—in resistant root microbiomes (Fig. 1F, G). Given that nitrate promotes *P. brassicae* spore germination and influence bacterial microbiota structure [27]. Based on these findings, we hypothesize that denitrifying bacteria may potentially play a role in plant disease resistance.

### Screening of antagonistic and denitrifying bacteria for suppression of *P. brassicae* resting spore germination

**Figure 2:**
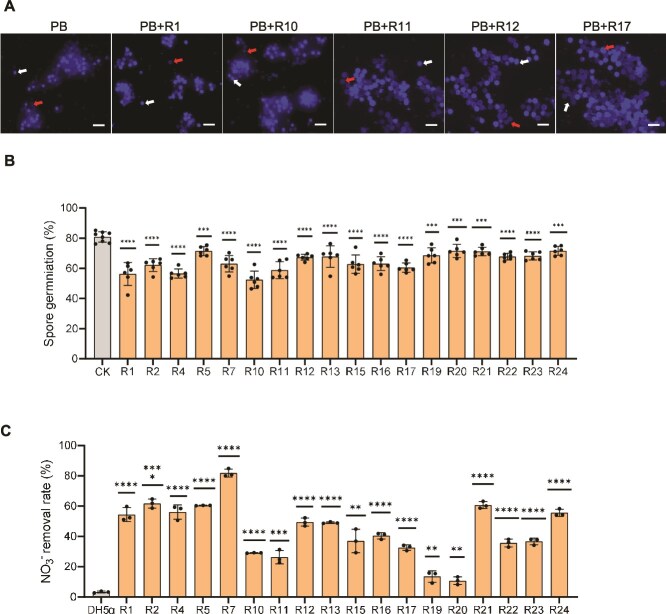
Screening of bacterial strains with dual functionality: Inhibition of resting spore germination and denitrification capacity. (A–B) Evaluation of *P. brassicae* resting spore germination inhibition: (A) Morphological assessment of sterilized *P. brassicae* resting spores after 3-day co-culture with bacterial fermentation broth, visualized by DAPI staining under UV (scale bar = 20 μm). Red arrows indicate the absence of fluorescence, corresponding to germinated resting spores; white arrows point to ungerminated spores. (B) Quantification of resting spore germination rates. Data represent mean ± SD (n = 6 biological replicates). Statistical significance was determined by two-tailed Student’s *t*-test. (C) Determination of bacterial nitrate removal rate using flow injection analysis. Bacteria were cultured in DM medium with KNO₃ as the sole nitrogen source (5 g sodium citrate, 0.2 g MgSO₄·7H₂O, 1 g KH₂PO₄, 1 g K₂HPO₄, 2 ml trace element solution, and 2 g K₂NO₃; pH adjusted to 7.2 and volume brought to 1 l with sterile water). Nitrate content was measured after 1 hour and 24 hours of incubation, and the nitrate removal rate was calculated. Data represent mean ± SD (n = 3 biological replicates). Statistical significance was determined by two-tailed Student’s *t*-test (^*^P < .05, ^**^P < .01, ^***^P < .001, ^****^P < .0001). The experiment was repeated twice with similar results.

To identify potential biocontrol agents against *P. brassicae*, we isolated 500 bacterial strains from the roots of clubroot-resistant rapeseed cultivars. Given that resting spore germination is the pathogen’s initial infection step and a critical target for disease control, we screened isolates for their ability to inhibit spore germination. Eighteen strains consistently suppressed resting spore germination under controlled conditions (Fig. 2A-B, Table S3). All 18 strains exhibited denitrification capacity, indicated by blue coloration in Giltay medium (Fig. S4). Flow injection analyzer (FIA) measurements confirmed nitrate removal activity, with strain R7 achieving the highest removal efficiency (80%) and strain R1 exceeding 50% (Fig. 2C). These validated strains were assembled into a synthetic microbial community, SynCom^18^. To further characterize their denitrification potential, we sequenced the genomes of three representative strains. All harbored genes encoding nitrate reductase and other N metabolism–related enzymes (Table S4). Phylogenetic analysis identified strain R1 as *Microbacterium* sp., R10 as *Pseudomonas* sp., and R11 as *Bacillus* sp. (Fig. S5).

### Denitrifying bacteria suppress clubroot disease

**Figure 3:**
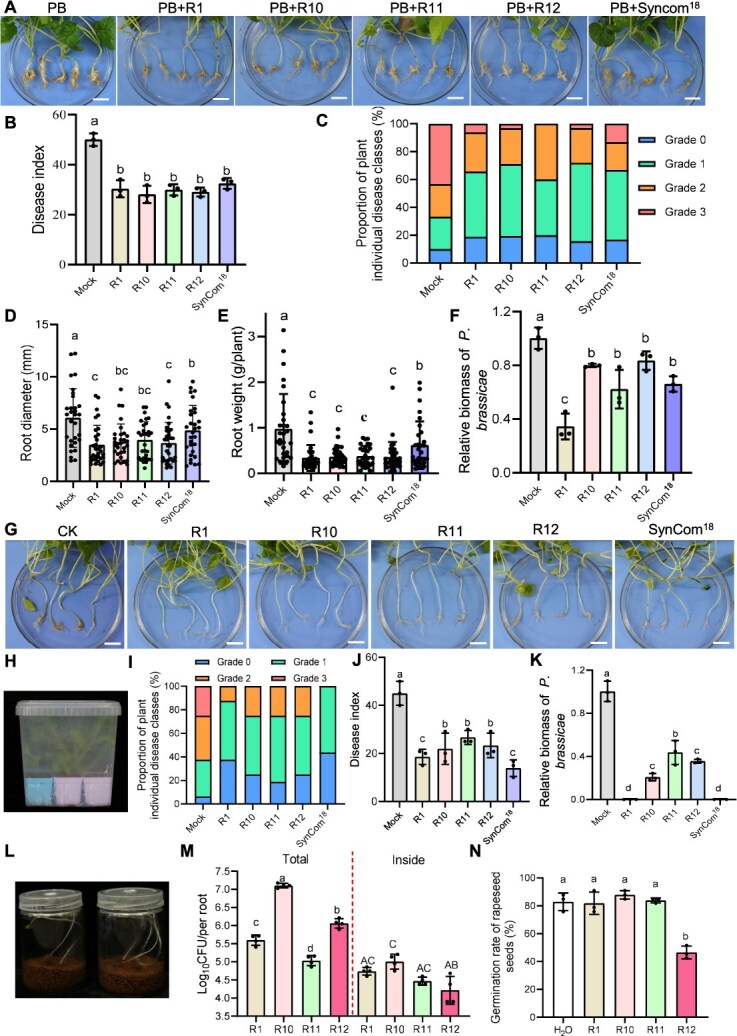
Control of clubroot disease by denitrifying bacteria and Syncom^18^ in pot experiments and gnotobiotic system. (A–F) Effects of denitrifying bacterial strains (R1, R10, R11, R12) and Syncom^18^ on rapeseed 30 days after *P. Brassicae* inoculation in pot experiment: (A) root gall phenotype (scale bar = 2 cm), (B) disease index, (C) percentage distribution of disease severity classes, (D) root diameter (root diameter is defined as the diameter at the thickest part of the root and is measured using a Vernier caliper), (E) root weight, (F) relative *P. brassicae* biomass. For disease index analysis (B), n = 30–32 biological replicates were randomly divided into three groups. The relative biomass of *P. brassicae* (F) is represented by the content of the *P. brassicae ACTIN* gene relative to that of the *B. napus GDI1* gene. Primer sequences are in Table S2 (G-N) effects of denitrifying bacterial strains (R1, R10, R11, R12) and Syncom^18^ treatment in a gnotobiotic system: (G) root gall phenotype (scale bar = 2 cm), (H) gnotobiotic system setup for clubroot disease assessment, (I) percentage distribution of disease severity classes, (J) disease index, and (K) relative *P. brassicae* biomass (quantified by qPCR) at 30 days post-inoculation. For disease index analysis, n = 32 biological replicates were randomly divided into three groups; for *P. brassicae* biomass, n = 3 biological replicates. (L-N) (L) Gnotobiotic system setup for the assessment of bacterial colonization in rapeseed roots and (M) root colonization rates (surface and endophytic) of rapeseed co-cultured with denitrifying bacterial strains (R1, R10, R11, R12) in sterile culture vessels for 14 days (n = 4). Statistical analysis as above. (N) Germination rate of surface-sterilized rapeseed seeds following 8 h treatment with bacterial suspension (OD_600_ = 0.4) and 6-day culture in Petri dishes (33 seeds per treatment, n = 3 biological replicates). Control received sterile water. Error bars show mean ± SD. Statistical significance was determined by one-way ANOVA with Duncan’s multiple range test (different letters indicate significant differences at *P* < .05).

To evaluate the biocontrol potential of denitrifying bacteria against *P. brassicae*, we conducted a rapeseed pot experiment and assessed disease symptoms 30 days post-inoculation. Treatments with SynCom^18^ and individual bacterial strains (R1, R10, R11, R12) significantly reduced root gall formation (Fig. 3A-F), with disease index significantly lower than those of the untreated controls (Fig. 3B-C). Additionally, root biomass and *P. brassicae* relative abundance significantly reduced in the bacterial treatment groups compared to the control (Fig. 3D-F). These findings suggest that these bacterial isolates and SynCom^18^ may have the potential as biocontrol agents for managing clubroot disease.

To evaluate the intrinsic biocontrol capacity of bacterial strains without interference from other microbes, we established a gnotobiotic system for rapeseed (Fig. 3G-K). At 28 days post-inoculation, root galls were prominent in control plants, whereas bacterial treatments significantly reduced or eliminated gall formation (Fig. 3G). Disease index, the proportion of severely affected plants, and *P. brassicae* biomass were all significantly lower in treated groups compared to controls (Fig. 3I-K). All tested denitrifying strains successfully colonized both root endospheres and surface (Fig. 3L-M). However, treatment of seeds with the R12 strain significantly reduced the seed germination rate, whereas R1, R10, and R11 did not affect germination rates. (Fig. 3N). These results suggest R1, R10, R11, and SynCom^18^ as promising candidates for field application in clubroot biocontrol.

### Denitrifying bacteria suppress clubroot disease and enhance yields under field conditions

**Figure 4:**
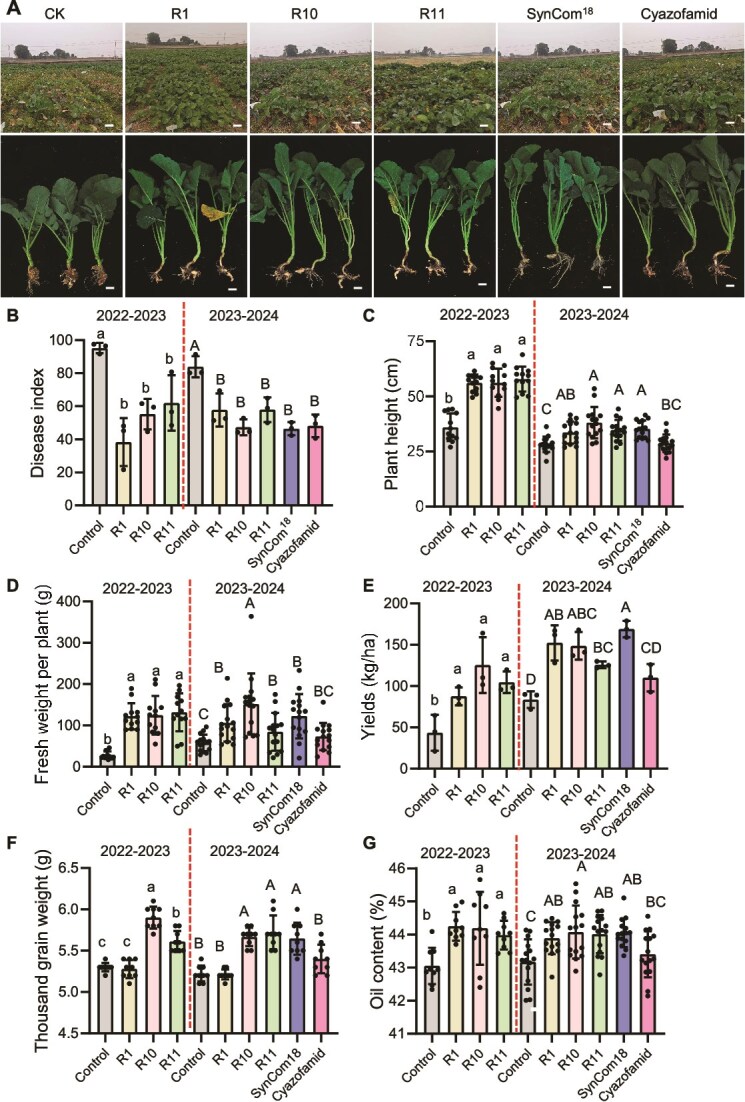
Field evaluation of clubroot disease control using denitrifying bacterial strains in rapeseed. (A) Phenotypic comparison of rapeseed plants with and without denitrifying bacterial strain treatment after 63 days (seedling stage, 2023–2024 growing season) in clubroot field trials (Zhijiang, Hubei Province, China). Top: Field plot overview (scale bar = 20 cm). Bottom: Three representative plants (scale bar = 10 cm). (B) Disease index assessed at 76 days (2022–2023 season) or 63 days (2023–2024 season) post-treatment. Each treatment included three replicate plots with 50 randomly selected rapeseed plants per plot. (C-D) Aboveground plant height (C) and fresh weight (D) measured after 100 days of bacterial treatment. From each plot, 4–5 representative plants were sampled (total n = 12–15 across three plots). (E-G) Yield parameters: (E) seed yield, (F) thousand-grain weight, and (G) oil content. Biological replicates: n = 3 for yield, n = 9 for grain weight, n = 9–15 for oil content. Data in B-G are presented as mean ± SD. Statistical significance was determined by one-way ANOVA with Duncan’s multiple range test (different letters indicate significant differences at *P* < .05).

Biocontrol strains often perform well under controlled conditions but fail to deliver consistent results in the field due to environmental variability. To evaluate the practical efficacy of denitrifying bacteria against *P. brassicae*, we conducted two consecutive field trials in Zhijiang City, Hubei Province, China—a site with a consistent annual clubroot incidence exceeding 80%. Treatments with strains R1, R10, R11, and SynCom^18^ significantly reduced root gall formation (Fig. 4A). Across both seasons, all treatments led to a consistent reduction in disease index, averaging 41.6% (Fig. 4B).

Agronomic performance was assessed at seedling, flowering, and maturity stages. At the seedling stage, treatments significantly improved plant height, fresh weight, leaf number, and stem diameter (Fig. 4C-D, S6). At flowering, enhanced plant height, stem diameter, and biomass were observed (Fig. S7A-E). At maturity, R10 increased effective branch number (both seasons), silique number on the main inflorescence (2022–2023), and total siliques per plant (2023–2024) (Fig. S7F-H). At harvest, all treatments significantly increased yield per hectare—91.8% (R1), 133.1% (R10), 94.5% (R11), and 102.0% (SynCom^18^)—relative to controls (Fig. 4E). Thousand-grain weight (Fig. 4F), seed size (Fig. S8), and oil content (Fig. 4G) also improved significantly. These findings demonstrate that denitrifying bacteria not only suppress clubroot disease under field conditions but also promote rapeseed growth, yield, and oil quality, supporting their potential as sustainable biocontrol agents.

### Increased N fertilization aggravates clubroot disease severity

**Figure 5:**
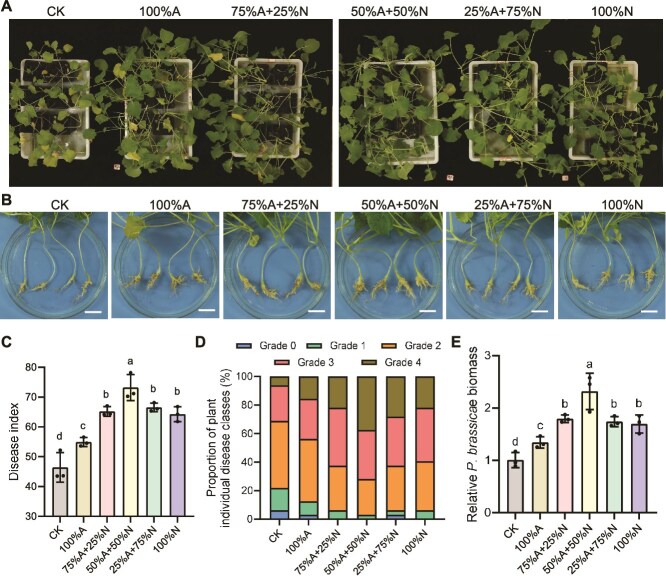
Effects of different nitrogen forms on clubroot disease development. (A) Shoot phenotype, (B) root gall phenotype, (C) disease index, (D) percentage of plants at each disease severity grade, and (E) relative *P. brassicae* biomass in infected roots (qPCR). Plants were inoculated with *P. brassicae* and grown for 28 days in pot experiments under a fixed total nitrogen supply (10 mM) with varying NH_4_^+^ and NO_3_^−^ ratios (A: ammonium; N: nitrate). Scale bars: 10 cm (A), 3 cm (B). Sample sizes: n = 32 biological replicates (randomized into three groups for C); n = 3 technical replicates (E). Data are mean ± SD. Significant differences (*P* < .05, one-way ANOVA with Duncan’s test) are denoted by lowercase letters.

N is essential for crop productivity, but excessive application can influence disease susceptibility. Rapeseed, known for its low N use efficiency [49], relies heavily on N fertilization; however, the relationship between N input and clubroot severity remains unclear. To assess this, we conducted pot experiments using Hoagland nutrient solutions with different N forms (NH₄^+^ or NO₃^−^), maintaining a total N concentration of 10 mM across treatments. In N-free conditions, plants exhibited poor growth and leaf chlorosis. By contrast, N application significantly improved shoot growth (Fig. 5A) but also exacerbated root gall formation (Fig. 5B). Disease index, maximum severity grades, and *P. brassicae* biomass in infected roots were all significantly higher in N-treated plants than in controls (Fig. 5C-E). These findings indicate that whereas N fertilization promotes growth, it concurrently increases susceptibility to clubroot, highlighting a trade-off between growth and disease resistance, mediated by N fertilization.

### Denitrifying bacteria reduce soil nitrate and enhance resistance to clubroot

**Figure 6:**
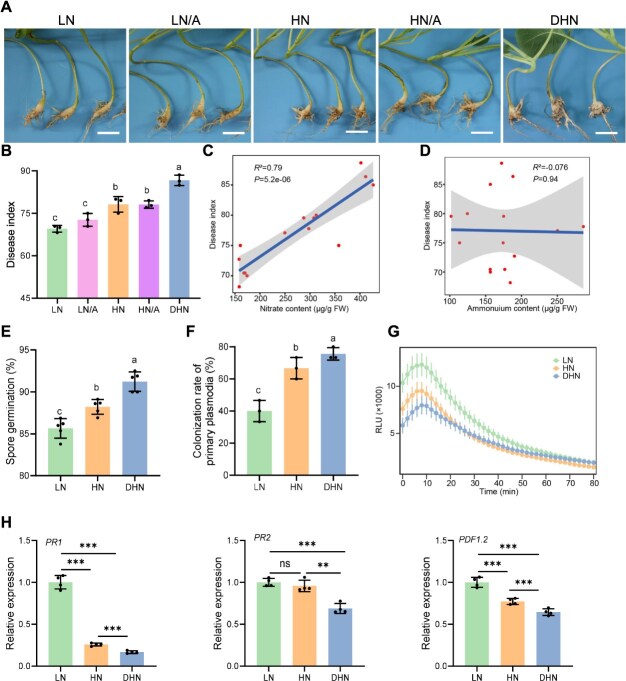
Nitrogen-dependent biocontrol efficacy against *P. brassicae* infection in rapeseed. (A-B) Phenotypic and pathological assessment 28 days post *P. brassicae* inoculation under varying nitrogen conditions: (A) Representative root morphology (scale bar = 3 cm), (B) disease index quantification. Nitrogen treatments: LN: 6 mM NO₃^−^, HN: 30 mM NO₃^—^N, DHN: 60 mM NO₃^−^, LN/A: 6 mM total N (NH₄^+^-N:NO₃^−^-N = 7:3), HN/A: 30 mM total N (NH₄^+^-N:NO₃^—^N = 7:3). Disease index evaluation: n = 32 biological replicates (randomized into 3 analytical groups). (C-D) Pearson correlation analyses between: (C) Disease index and soil NO₃^-^-N content, (D) Disease index and soil NH₄^+^-N content. (E-F) effects of different nitrate nitrogen levels on the germination rate of resting spores (E) and the formation rate of primary plasmodia within root hairs (F). Sterilized resting spores were cultured in Hoagland nutrient solutions with varying nitrate nitrogen concentrations in the dark for 2–3 days. Germination rate of resting spores was determined using the DAPI staining method. Germination rate of resting spores (%) = (number of germinated resting spores / Total number of resting spores) × 100%. n = 5. Rapeseed plants hydroponically grown for 14 days were treated with different nitrate nitrogen concentrations for two days. Two days after inoculation with *P. brassicae*, roots were stained with trypan blue, randomly cut into 1-cm segments, and grouped into sets of 15 segments. Three such groups were examined per sample. Root hair infection, specifically the formation of primary plasmodia, was observed under a microscope. (G–H) Detection of ROS (G) and expression of immune-related genes (H) in rapeseed under different nitrate nitrogen levels. Rapeseed plants were hydroponically grown for 7 days and then cultured in Hoagland nutrient solutions with varying nitrate nitrogen concentrations for 3 days. Root segments (0.5 cm in length) were excised and treated with 50 ng/ml chitin for ~90 minutes, followed by ROS burst detection (n = 10). RNA was extracted from leaves, and the expression of *PR1*, *PR2*, and *PDF1.2* genes was measured using qPCR (n = 4). Primer sequences are listed in Table S2. Data in B, E and F are presented as mean ± SD. Statistical significance was determined by one-way ANOVA with Duncan’s multiple range test (different letters indicate significant differences at *P* < .05). Data in G were determined by two-tailed Student’s *t*-test, with ^*^  *P* < .05, ^**^P < .01, ^***^P < .001.

To investigate how N forms and availability affect clubroot development, we conducted a factorial experiment using rapeseed under varying N regimes during *P. brassicae* infection. Treatments included: (i) low nitrate (LN), (ii) medium nitrate (HN), (iii) high nitrate (DHN), and ammonium-nitrate mixes (LN/A and HN/A). At 28 dpi, increased nitrate levels significantly aggravated root galling and disease severity (Fig. 6A-B). Soil nitrate content was strongly correlated with disease index (*R*^2^ = 0.79, *P* = 5.2e-06; Fig. 6C, Fig. S9A-B), whereas ammonium showed no significant relationship (*R*^2^ = 0.076, *P* = .94; Fig. 6D, Fig. S9C). Treatments with strains and SynCom^18^ consistently reduced disease severity (Fig. S9A) and significantly decreased rhizosphere soil nitrate concentrations without altering ammonium levels (Fig. S9B, C). To elucidate the mechanism by which nitrate nitrogen influences the pathogenicity of *P. brassicae*, we conducted verifications from both the pathogen and plant perspectives. Firstly, in terms of the development of *P. brassicae*, high nitrate nitrogen treatment was found to be more conducive to the germination of resting spores and the formation of primary plasmodia in root hairs compared to low nitrate nitrogen treatment (Fig. 6E, F). Additionally, high nitrate nitrogen treatment suppressed the burst of ROS and the expression of immune-related genes *PR1*, *PR2*, and *PDF1.2* relative to low nitrate nitrogen treatment (Fig. 6G-H). These results suggest that denitrifying bacteria and SynCom^18^ may enhance plant disease resistance by reducing nitrate nitrogen levels in the rhizosphere soil, thereby inhibiting the development of *P. brassicae* and strengthening plant immunity.

### Resistant cultivars recruit denitrifying bacteria via aesculetin secretion to combat *P. brassicae* infection

**Figure 7:**
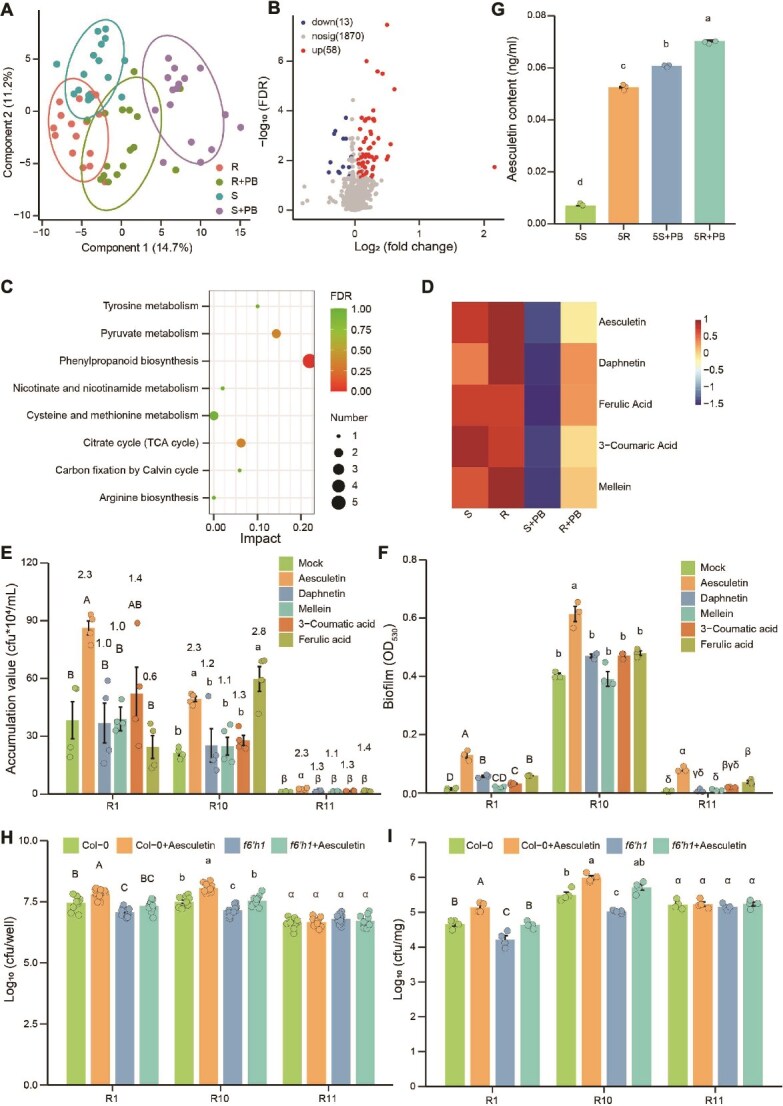
Comparison of rhizosphere metabolomics between clubroot resistant cultivars (Huayouza 62R and Huashuang 5R) and susceptible cultivars (Huayouza 62 and Huashuang 5) after infection with *P. brassicae.* (A) OPLS-DA analysis of rapeseed rhizosphere non-target metabolomics with a sample size of n = 8. (B) Volcano plot of rapeseed rhizosphere metabolomics differences between clubroot-resistant cultivars (Huayouza 62R and Huashuang 5R) and susceptible cultivars (Huayouza 62 and Huashuang 5) inoculated with *P. brassicae* (VIP ≥ 1 and FDR < 0.05). (C) KEGG pathway analysis of 58 metabolites enriched in clubroot-resistant cultivars after *P. brassicae* infection. (D) Heatmap of five metabolites in the phenylpropanoid biosynthesis. (E) Chemotactic response of denitrifying bacteria R1, R10 and R11 towards five metabolites. Numbers on top of each bar indicate the relative chemotactic indexes (RCI). RCI ≥ 2 indicates a strong chemotactic response. n = 4. (F) Five metabolites affect R1, R10 and R11 biofilm formation. S: Huayouza 62 and Huashuang 5, R: Huayouza 62R and Huashuang 5R, PB: *P. brassicae*. n = 3. (G) Quantification of aesculetin content in root exudates of Huashuang 5 and Huashuang 5R inoculated with or without *P. brassicae* using HPLC, n = 3. (H–I) Detection of bacterial colonization rates using a sterile system. Col-0 and *f6’h1* mutants were grown in 24-well plates for 12 days and then inoculated with R1, R10, and R11 strains, respectively, with or without 100 μM exogenous aesculetin. After two days, bacterial colonization rates in the rhizosphere (H, n = 10) and roots (I, n = 4; three plants pooled as one sample) were determined. Data in E, F, G, H, and I are presented as mean ± SD. Statistical significance was determined by one-way ANOVA with Duncan’s multiple range test (different letters indicate significant differences at *P* < .05).

To explore the disease resistance mechanisms of the clubroot-resistant cultivars Huayouza 62R and Huashuang 5R, we conducted untargeted metabolomic sequencing of root exudates from these resistant cultivars and their susceptible parents (Fig. 7A). Orthogonal least partial squares discriminant analysis (OPLS-DA) revealed distinct metabolic responses to *P. brassicae* between susceptible and resistant cultivars. Fifty-eight metabolites were significantly enriched in the rhizosphere of *P. brassicae*-inoculated resistant cultivars (Fig. 7B). Pathway analysis identified five metabolites from the phenylpropanoid biosynthesis pathway (Fig. 7C): aesculetin, daphnetin, ferulic acid, 3-coumaric acid, and melletin (Fig. 7D). Subsequently, we evaluated the effects of these five metabolites on chemotaxis and biofilm formation in denitrifying bacterial strains R1, R10, and R11 (Fig. 7E-F), only aesculetin exhibited strong chemotactic activity (RCI > 2) and significantly enhanced biofilm formation in these strains. The content of aesculetin in the rhizosphere of plants was quantitatively determined using the HPLC method (Fig. 7G). The results showed that the resistant variety exhibited significantly higher levels than the susceptible variety, both under conditions of inoculation with *P. brassicae* and without inoculation. We conducted bacterial recruitment assays using wild-type Col-0 and mutants defective in coumarin biosynthesis or secretion (*f6’h1*) [30] (Fig. S10, Fig. 7H-I). Exogenous addition of aesculetin significantly enhanced the colonization of strains R1 and R10 in both the rhizosphere and root of Col-0 plants. Regardless of exogenous aesculetin application, the colonization of R1 and R10 in the rhizosphere and root of Col-0 plants was significantly higher than that in *f6’h1* mutant plants. Our findings demonstrate that resistant cultivars, upon pathogen challenge, secrete aesculetin via root exudates to recruit denitrifying bacteria, thereby aiding in the defense against *P. brassicae* infection.

## Discussion

**Figure 8:**
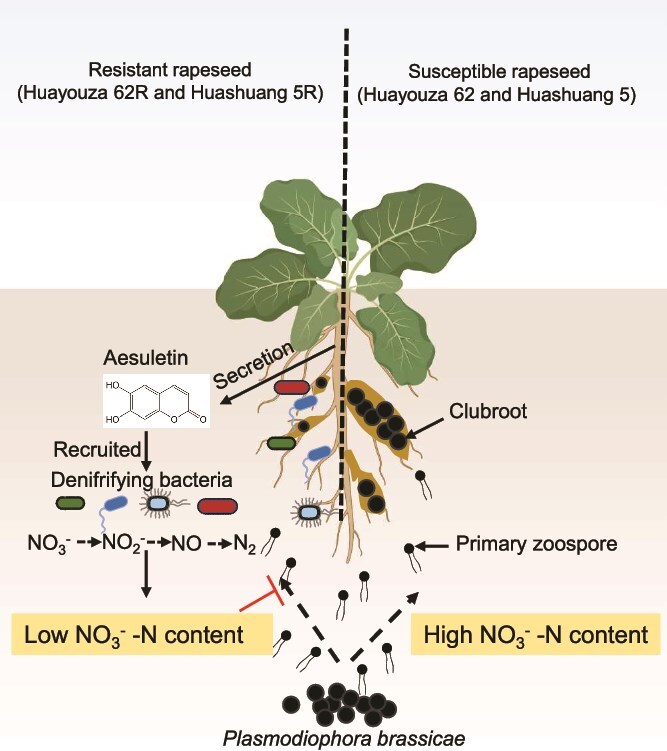
Working model for resistant rapeseed (Huayouza 62R and Huashuang 5R) to regulate rapeseed resistance to *P. brassicae*. After *P. brassicae* infection, the plant secretes aesculetin, which recruits denitrifying bacteria, to reshape the rhizosphere microbiome and deplete the soil nitrate through denitrification. Both denitrifying bacteria and the low nitrate content suppress *P. brassicae* resting spores’ germination and help with resistance to *P. brassicae*.

This study reveals a previously unrecognized mechanism of microbiota—mediated resistance to clubroot disease: suppression of *P. brassicae* through microbial denitrification (Fig. 8). We demonstrate that following *P. brassicae* infection, resistant cultivars develop a distinct microbial community structure compared to susceptible cultivars. The key mechanism involves the secretion of the coumarin derivative aesculetin, which recruits denitrifying bacteria. These bacteria enhance rapeseed resistance to clubroot by reducing rhizosphere nitrate-nitrogen levels via denitrification and suppressing *P. brassicae* germination. The denitrifying bacteria exhibit robust biocontrol efficacy in both pot and field trials, highlighting their potential for practical application. Microbiome analysis is commonly used to identify key disease-suppressing microorganisms. For instance, omics analysis of disease-resistant and susceptible rice seeds revealed tha*t Sphingomonas melonis* accumulates and is trans generationally transmitted in resistant seeds, conferring disease resistance via anthranilic acid production [50]. Similarly, rhizosphere microbiome and metagenome analysis of tomato varieties identified the Flavobacterium strain TRM1, which effectively suppresses bacterial wilt [51]. In this study, our strategy is similar, and we have successfully screened for biocontrol bacteria that help suppress clubroot disease. These results suggest that using microbiome analysis to guide the discovery of disease-suppressing microbes will be a common and effective approach.

Breeding for resistance remains a cornerstone of clubroot management. At least 32 CR loci have been mapped across *Brassica* species, though only three genes—*CRa*, *CRb*, and *Crr1a*—have been cloned, all encoding TIR-NB-LRR-type R proteins. Despite this progress, the molecular mechanisms by which these R proteins mediate resistance remain poorly understood [52–54]. The cultivars used in this study, Huayouza 62R and Huashuang 5R, carry the *CRb* and *PbBa8.1* loci, respectively—both of which may share allelic relationships with known *CR* genes [11, 12, 55]. These resistant materials were obtained through multiple rounds of hybridization and backcrossing. During molecular marker-assisted selection, the *PbBa8.1* resistance locus was used as the core breeding objective. However, the *PbBa8.1* loci are located within a 2.9 Mb region on chromosome A08. Due to the large size of this fragment, it is currently impossible to precisely determine how many resistance genes are present. It is generally believed that multiple resistance genes exist in this region, but the specific gene responsible for the resistance function remains unidentified. Despite their genetic basis in R gene-mediated resistance, these cultivars exhibited distinct root microbiomes compared to susceptible lines, suggesting that microbial composition contributes to their overall resistance phenotype. This observation aligns with emerging evidence that R genes may influence microbiome assembly. The concept of “M genes” —genes that shape (Ms genes) or respond to microbiota (Mr genes)—has been proposed to describe plant genomic components that mediate host-microbiota interactions [56]. Integrating R gene- and M gene-mediated strategies could enable more durable disease control by combining genetic and microbiome-based resistance [57–59]. However, the mechanistic overlap between these gene types and their influence on microbial recruitment remain poorly understood, and further dissection of these interactions will be critical for future breeding strategies.

Coumarins, such as scopolin, scopoletin, aesculin, aesculetin, and isofraxidin, are synthesized through the phenylpropanoid pathway [60]. They accumulate significantly in the roots and root exudates of iron-deficient plants, where they are involved in the mobilization and uptake of iron [61]. Among them, *MYB72* specifically regulates the excretion of scopoletin, a coumarin that is an iron-mobilizing phenolic compound. Scopoletin also has selective antimicrobial activity, which helps to shape the root-associated microbial community for the well- being of plants [30]. T-DNA insertion mutants of *F6’H1* showed severe reductions in scopoletin and were defective in coumarin biosynthesis [62]. Aesculetin (6,7-dihydroxycoumarin), a bioactive coumarin derivative, is known to function as a defense compound in plants and animals, with reported pharmacological properties such as anti-inflammatory, antioxidant, and antitumor effects [63–65]. In this study, we have confirmed that disease-resistant varieties secrete more aesculetin to recruit denitrifying bacteria R1 and R10 (Fig. 7). This recruitment of R1 and R10 to colonize plant roots was further validated using the *f6’h1* mutant, which is defective in coumarin biosynthesis. However, denitrifying bacteria R11 cannot be recruited by aesculetin (Fig. 7H-I), suggesting that not all denitrifying bacteria are recruited. Nevertheless, the specific recognition mechanism remains unclear. Resistant cultivars exhibit elevated aesculetin levels even in the absence of pathogen challenge (Fig. 7G). This suggests that aesculetin secretion is at least partly constitutive, rather than being strictly induced by *P. brassicae*. This finding has important implications for interpretation: aesculetin should be viewed as a predisposing host trait that shapes microbiome assembly and confers enhanced resilience, rather than solely as an inducible defence signal. Other studies have shown that coumarins can negatively affect biofilm formation and the virulence of pathogenic bacteria in different systems [66, 67]. Our research results show that it enhances the biofilm formation of denitrifying bacteria (Fig. 7F). Whether aesculetin can directly inhibit the growth and colonization of pathogens needs further testing.

Biocontrol approaches have received increasing attention as sustainable alternatives to chemical control [68]. Whereas numerous bacteria and fungi have shown efficacy against *P. brassicae* [69], field performance often remains inconsistent due to environmental complexity, native microbial competition, and application constraints. In this study, denitrifying bacteria demonstrated reproducible biocontrol effects across pot, gnotobiotic, and field systems, including improved agronomic traits and yield stability. During the 2024 freezing rain event in Hubei Province, treated plants still produced higher yields, underscoring the robustness of this approach. Chemical fungicides such as fluazinam and cyazofamid are currently registered for clubroot control in China [3]. However, in our trials, cyazofamid reduced disease severity at early stages but negatively impacted root development and failed to improve flowering, yield, or quality. In contrast, denitrifying strains and SynCom^18^ not only suppressed disease but enhanced multiple agronomic parameters. These findings support the superior field performance and agronomic compatibility of biological control based on denitrification. SynCom^18^ was tested only in one field season due to time constraints, showing performance comparable to single strains but without a consistent advantage. Its preparation was also cumbersome for field application. Therefore, two-strain combinations were explored. Strains R1, R10, and R11 showed good biocontrol in both pot and field experiments. Inhibition assays on plates and in co-culture revealed antagonism between some strains (Fig. S11A–F), possibly explaining why SynCom^18^ did not outperform single strains. Based on the co-culture results, six two-strain combinations were selected. All combinations, single strains and SynCom^18^ inhibited *P. brassicae* resting spores’ germination (Fig. S11G). Pot experiments showed that R10 + R19 and R11 + R19 outperformed their respective single strains (Fig. S11H–J). Although these results are from a single pot experiment and require field validation, they indicate that SynCom^18^’s potential for optimization. Additionally, both single bacteria and SynCom^18^ promoted rapeseed growth in the absence of pathogens (Fig. S12).

N availability is a key determinant of plant-pathogen interactions. Rapeseed is particularly N-demanding as its N use efficiency is low, requiring 8.8–11.3 kg N per 100 kg of seed, significantly more than cereals [49, 70]. Our results indicate that excessive nitrate, though beneficial for growth, exacerbates susceptibility to *P. brassicae*. This confirms earlier findings that nitrate promotes spore germination and can alter microbial community structure [22]. The observed correlation between soil nitrate and disease severity (*R*^2^ = 0.79, *P* = 5.2e-06) highlights nitrate as a modifiable factor in disease risk. Denitrifying bacteria offer a unique solution by decoupling nitrate availability from plant nutrition. In our study, inoculation with denitrifying strains reduced soil nitrate by ~39.4%, without affecting ammonium levels (Fig. S9). High nitrate nitrogen levels more readily promote the germination of dormant spores and the formation of primary plasmodia, whereas suppressing the plant’s ROS burst and the expression of immune-related genes compared to lower nitrate nitrogen levels (Fig. 6G, H). These bacteria effectively suppressed disease under both low and high nitrate conditions, providing a flexible tool for field application. Beyond disease suppression, the enhanced yield and quality traits further support their value in integrated crop management. Regarding the mechanism of suppression, we found that these denitrifying bacteria can reduce rhizosphere nitrate levels, and their secretions inhibit the germination of *P. brassicae* resting spores (Fig. 2, Fig. S9). Beyond these pathways, it is possible that other mechanisms also contribute to pathogen suppression—e.g. potential antagonistic interactions with *P. brassicae* or the induction of plant resistance responses.

In the future, in addition to controlling clubroot disease by combining judicious nitrogen fertilization—optimizing the nitrate-to-ammonium ratio—with microbial inoculants that deplete excess soil nitrate, it may also be possible to enhance plant resistance to clubroot through approaches such as breeding specific genotypes capable of constitutive aesculetin secretion and regulating the assembly of nitrate-associated microbial communities.

## Supplementary Material

Figure_S-1_wrag061

Table_S-1_wrag061

## Data Availability

The 16S rRNA gene sequencing data have been deposited in the NCBI short-read archive and can be accessed via the following DOI: https://identifiers.org/ncbi/insdc.sra:PRJNA1254883. The raw genomic sequencing data of strains R1, R10, and R11 are available in the NCBI SRA repository. These data can be accessed using the DOI: https://identifiers.org/ncbi/insdc.sra:PRJNA1269227. The metabolomics data have been deposited in the OMIX, China National Center for Bioinformation/Beijing Institute of Genomics, Chinese Academy of Sciences (https://ngdc.cncb.ac.cn/omix: accession PRJCA049529).
