# Supplementary material for: Aesculetin-mediated recruitment of denitrifying microbiota by resistant rapeseed suppresses Plasmodiophora brassicae via nitrate depletion
Source: ISME J. 2026 May 26;20(1):wrag061. doi: 10.1093/ismejo/wrag061 (PMC13215590; doi:10.1093/ismejo/wrag061)
Supplement: Figure_S-1_wrag061 [file figure_s-1_wrag061.pdf]

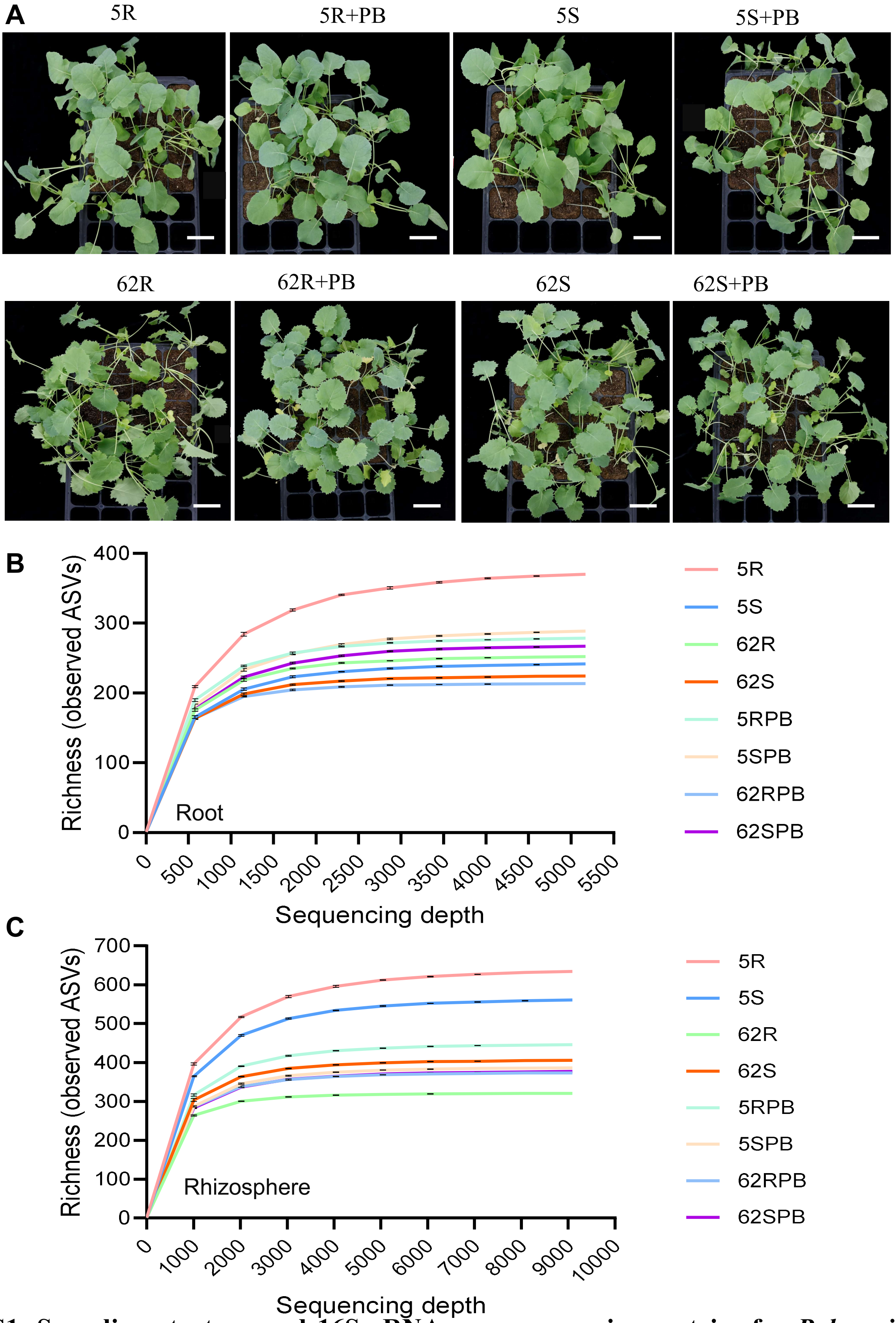

Figure S1. Sampling strategy and 16S rRNA gene sequencing metrics for *P. brassicae* -infected rapeseed cultivars.

(A) Aboveground phenotypes of clubroot-resistant (Huashuang 5R [5R], Huayouza 62R [62R]) and susceptible (Huashuang 5 [5S], Huayouza 62 [62S]) cultivars at 29 days post-inoculation. +PB: *P. brassicae* -inoculated; −PB: non-inoculated controls. (B–C) Rarefaction curves demonstrating sequencing depth sufficiency for microbial community analysis in: (B) Root endospheres and (C) Rhizosphere soil. Data representation: Mean ± SD (n = 7-8 biological replicates per group). Sequencing platform: Illumina NovaSeq 6000 System (2×250 bp paired-end). Bioinformatics: QIIME2-processed with 100% ASV clustering threshold.

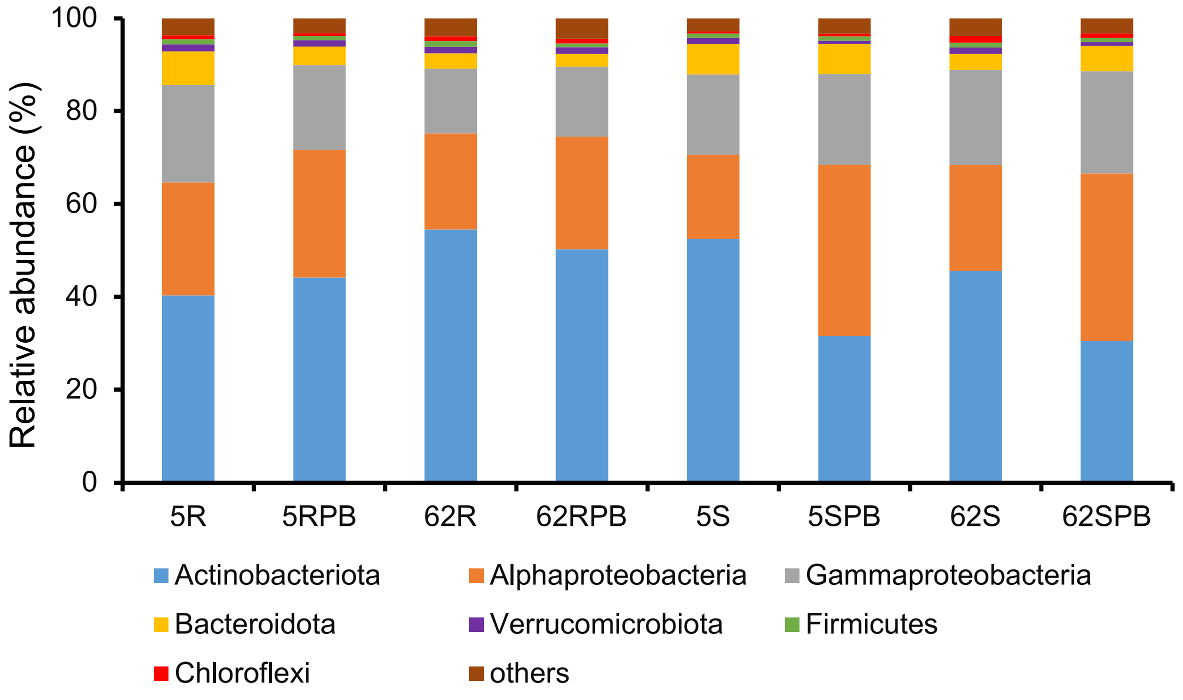

Figure S2. Bacterial community composition at the phylum level in rapeseed roots under *P. brassicae* infection. 16S rRNA gene sequencing reveals the relative abundance of root-associated bacterial phyla in: clubroot-resistant cultivars (5R, 62R) and clubroot-susceptible cultivars (5S, 62S).

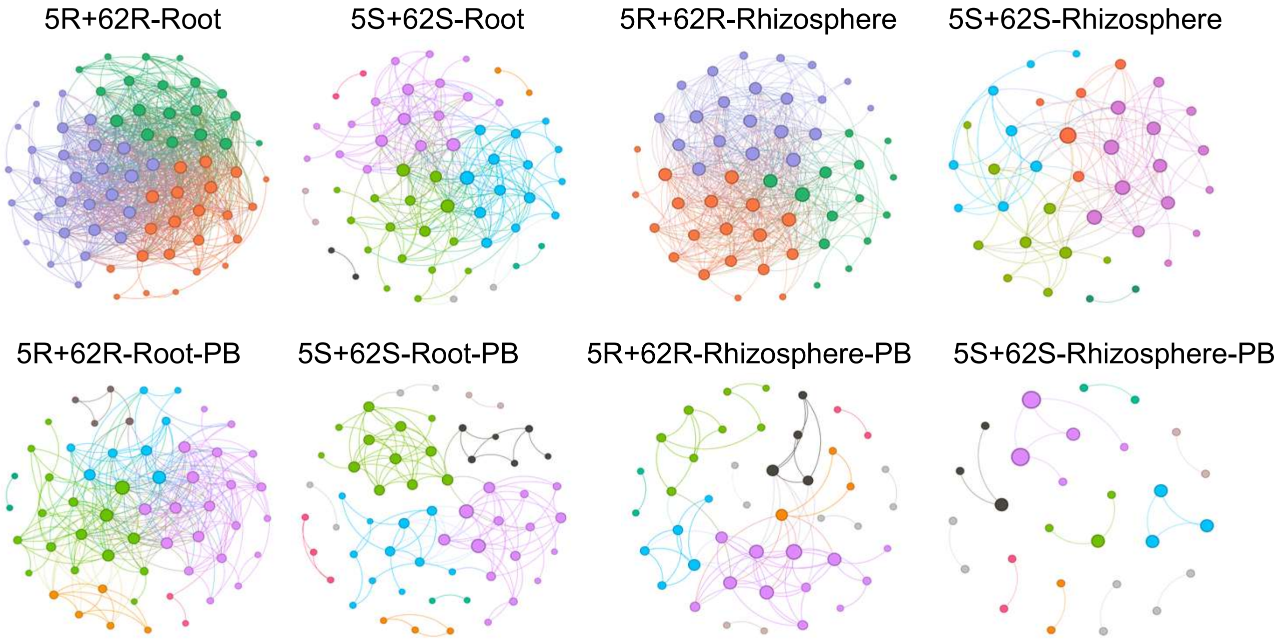

Figure S3. Co-occurrence network analysis comparing clubroot-resistant and susceptible cultivars. Upper panels: uninoculated controls; lower panels: *P. brassicae*-inoculated samples. Sample sizes: n = 7-8 biological replicates for root and rhizosphere microbiota analyses. The size of each node is determined by the degree of the node, and the colors of the nodes represent different network modules. Root samples of resistant cultivars contained 69 nodes and 928 edges (compared to 60 nodes and 253 edges in susceptible roots), while their rhizosphere soil showed 54 nodes and 543 edges (versus 38 nodes and 133 edges in susceptible rhizosphere). Following *P. brassicae* inoculation, although network complexity decreased across all samples, resistant cultivars maintained greater network stability and connectivity: their root systems retained 57 nodes and 295 edges (versus 58 nodes and 125 edges in susceptible roots), and rhizosphere networks contained 44 nodes with 80 edges (compared to 28 nodes and 19 edges in susceptible rhizosphere).

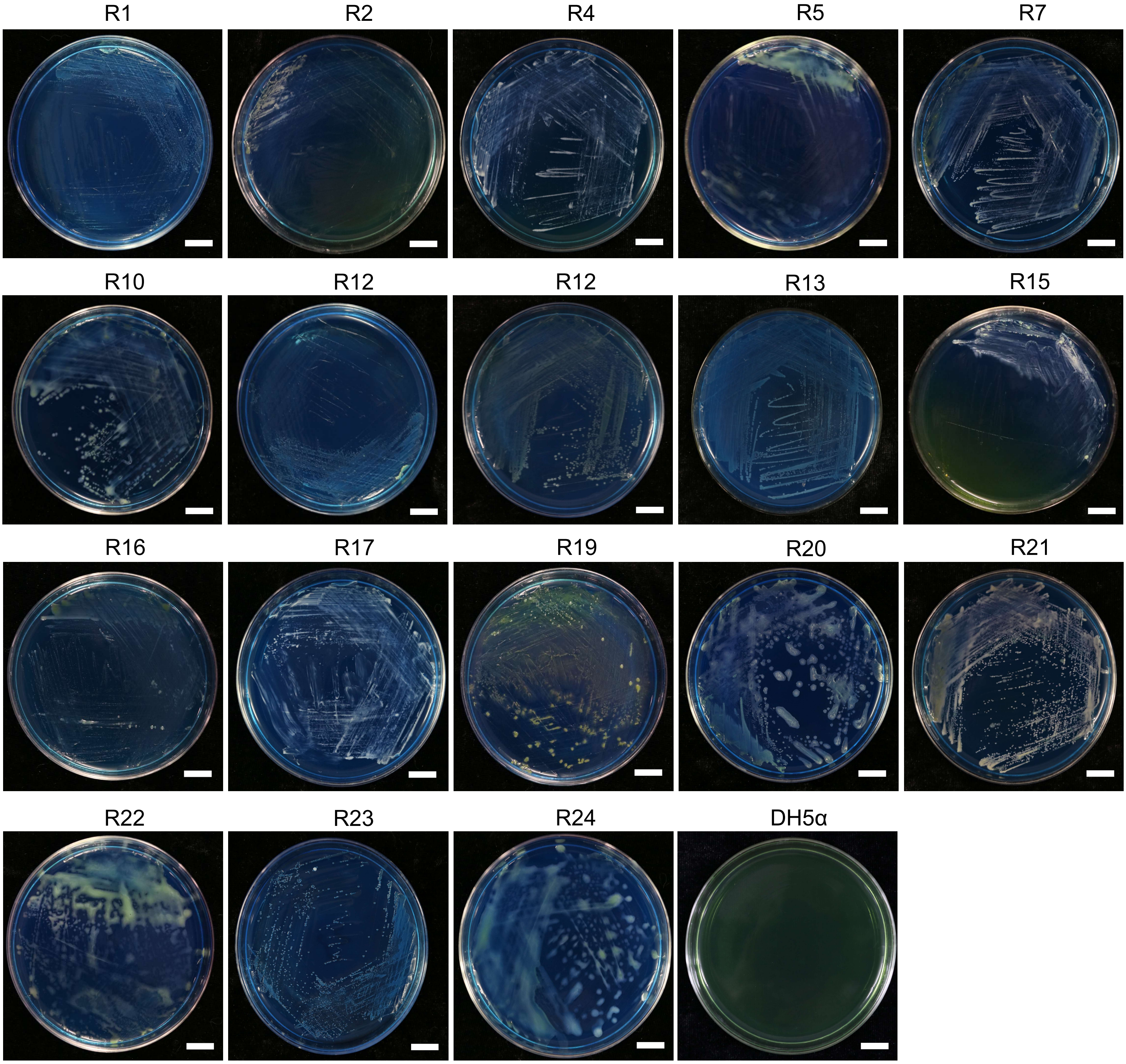

Figure S4. Eighteen denitrifying bacterial strains were isolated on Giltay medium, with denitrification activity indicated by blue coloration. *E. coli* DH5α served as the negative control. Scale bar = 1.5 cm.

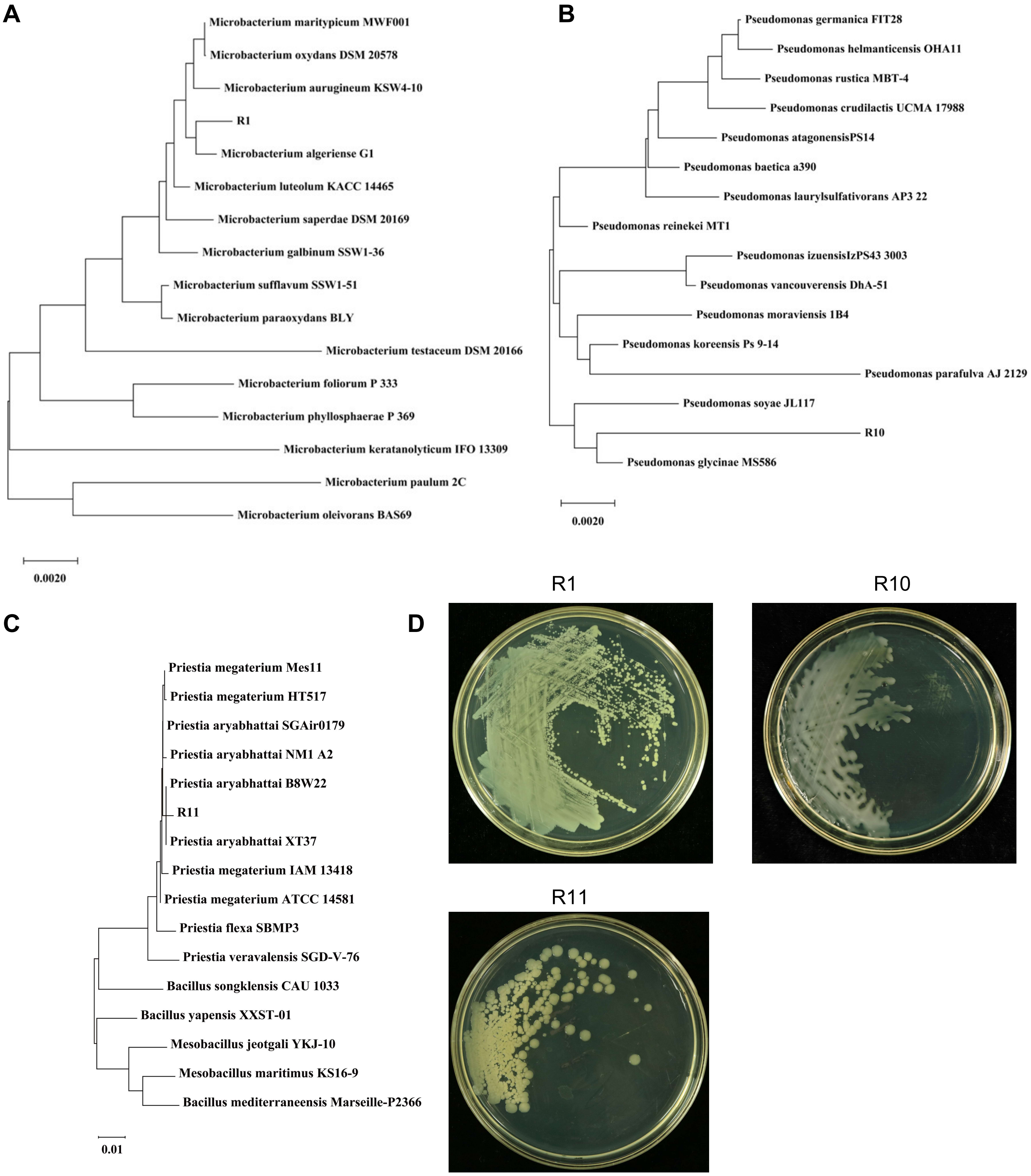

Figure S5. Phylogenetic tree of R1, R10 and R11 strain identification. Phylogenetic evolutionary tree of R1 (A), R10 (B) , R11(C) and phenotype of strains on R2A agar after 24-48 hours of incubation at 30°C(D). Phylogenetic evolutionary tree was constructed using the full length of 16S rRNA.

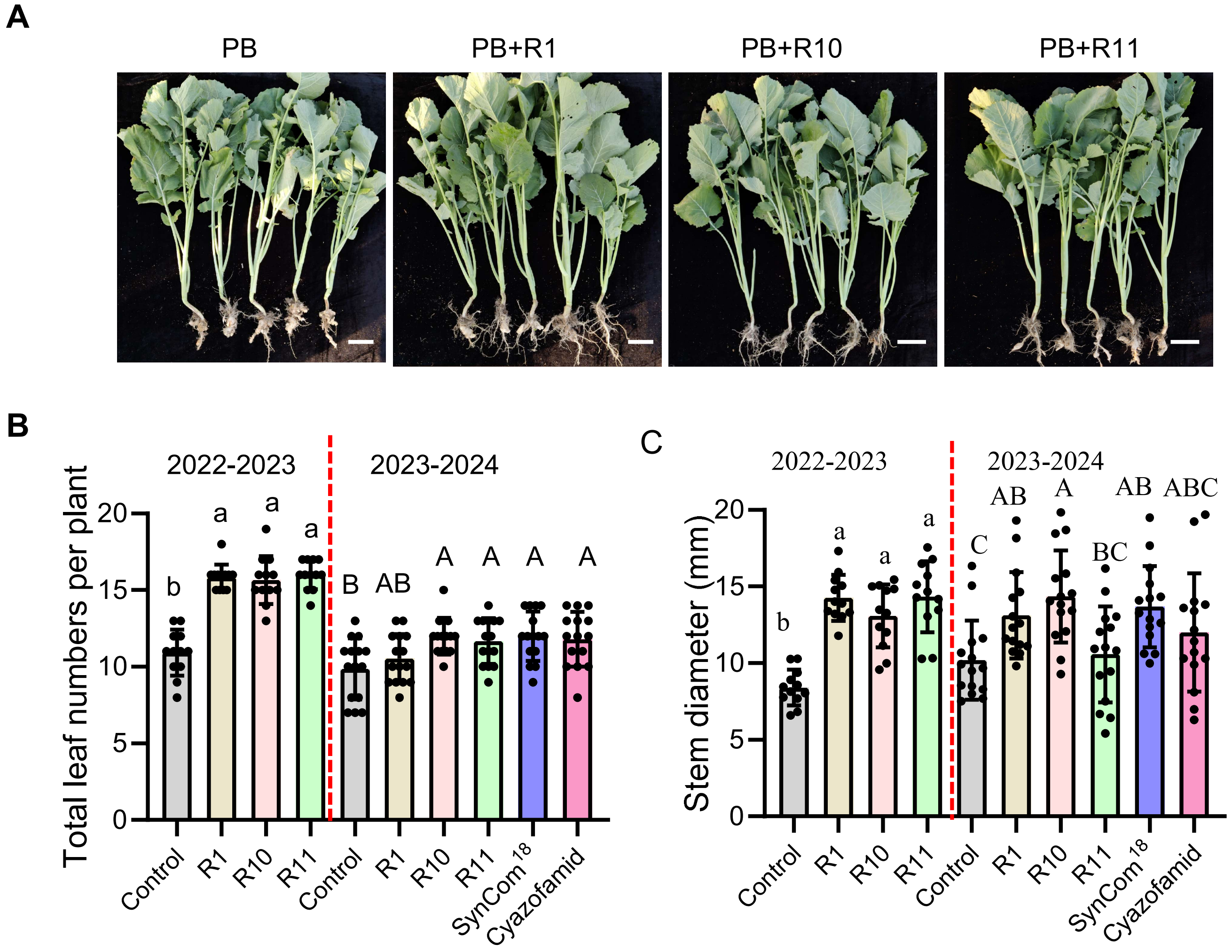

Figure S6. Agronomic trait statistics of field-grown rapeseed treated with denitrifying bacteria for clubroot disease control at seedling stage.

(A) Phenotypic comparison of rapeseed plants with and without denitrifying bacterial strain treatment after 76 days (seedling stage, 2022–2023 growing season) in clubroot field trials.

(B) Number of leaves per plant and (C) stem diameter measured 100 days after denitrifying bacterial treatment at the seedling stage. From each plot, 4-5 representative plants were sampled, total n=12-15 across three plots. Data in B, C are presented as mean ± SD. Statistical significance was determined by one-way ANOVA with Duncan's multiple range test (different letters indicate significant differences at $P<0.05$).

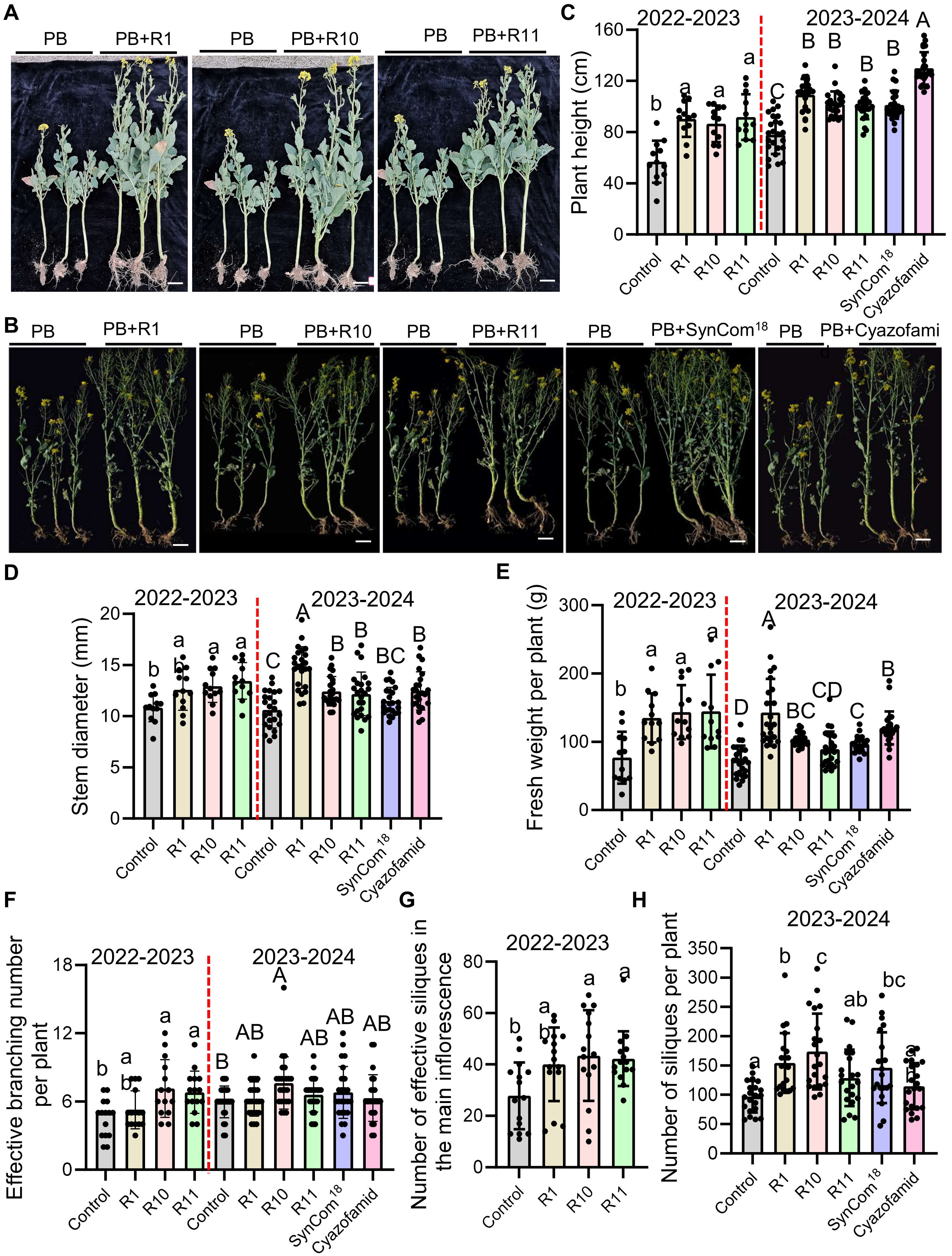

A
PB
PB+R1
PB
PB+R10
PB
PB+R11
B
PB
PB+R1
PB
PB+R10
PB
PB+R11
PB
PB+SynCom18
PB
PB+Cyazofami
d
C
2022-2023
2023-2024
Plant height (cm)
Control
R1
R10
R11
Control
R1
R10
R11
SynCom 18
Cyazofamid
D
2022-2023
2023-2024
Stem diameter (mm)
E
2022-2023
2023-2024
Fresh weight per plant (g)
F
2022-2023
2023-2024
Effective branching number per plant
G
2022-2023
Number of effective siliques in the main inflorescence
H
2023-2024
Number of siliques per plant

Figure S7. Agronomic trait analysis of field-grown rapeseed treated with denitrifying bacteria for clubroot disease control at flowering and mature stages.

(A–B) Phenotypic comparison of rapeseed plants with and without denitrifying bacterial strain treatment at 176 days (flowering stage, 2022–2023 growing season, A) or 181 days (flowering stage, 2023–2024 growing season, B). (C–E) Plant height (C), stem diameter (D), and fresh weight (E) measured at the flowering stage. (F–H) Effective branching number per plant (F), number of effective siliques in the main inflorescence (G), and total silique number per plant (H) measured at the mature stage. For panels C–H, biological replicates (n = 12–24) were analyzed. Data represent mean ± SD. Significant differences ($P < 0.05$) were determined by one-way ANOVA followed by Duncan's multiple range test (different lowercase letters indicate statistical significance).

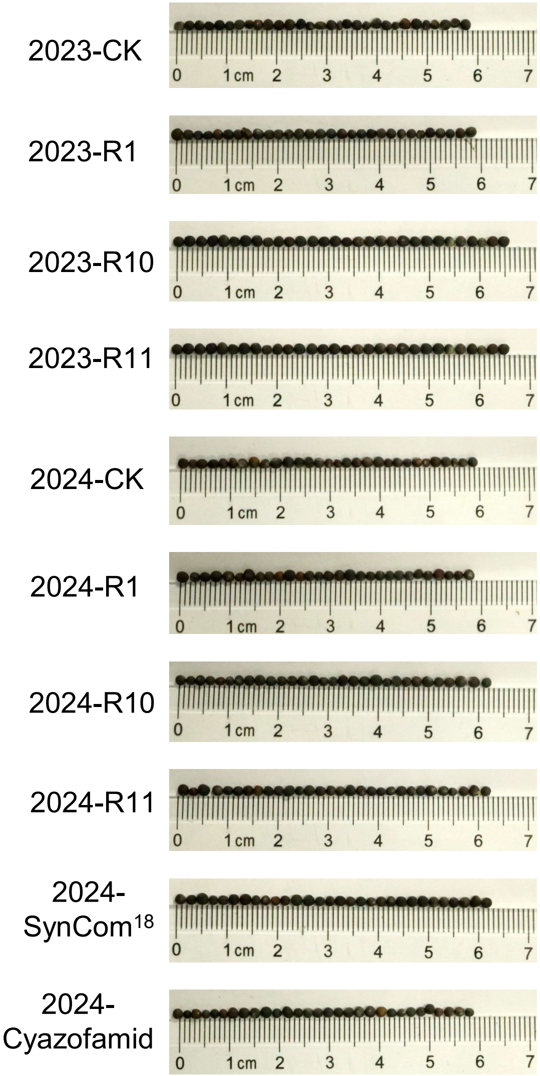

Figure S8. Seed phenotype. Phenotypic characterization of 30 randomly selected dried rapeseed seeds from field-grown plants treated with denitrifying bacteria and Syncom[18] for clubroot disease control.

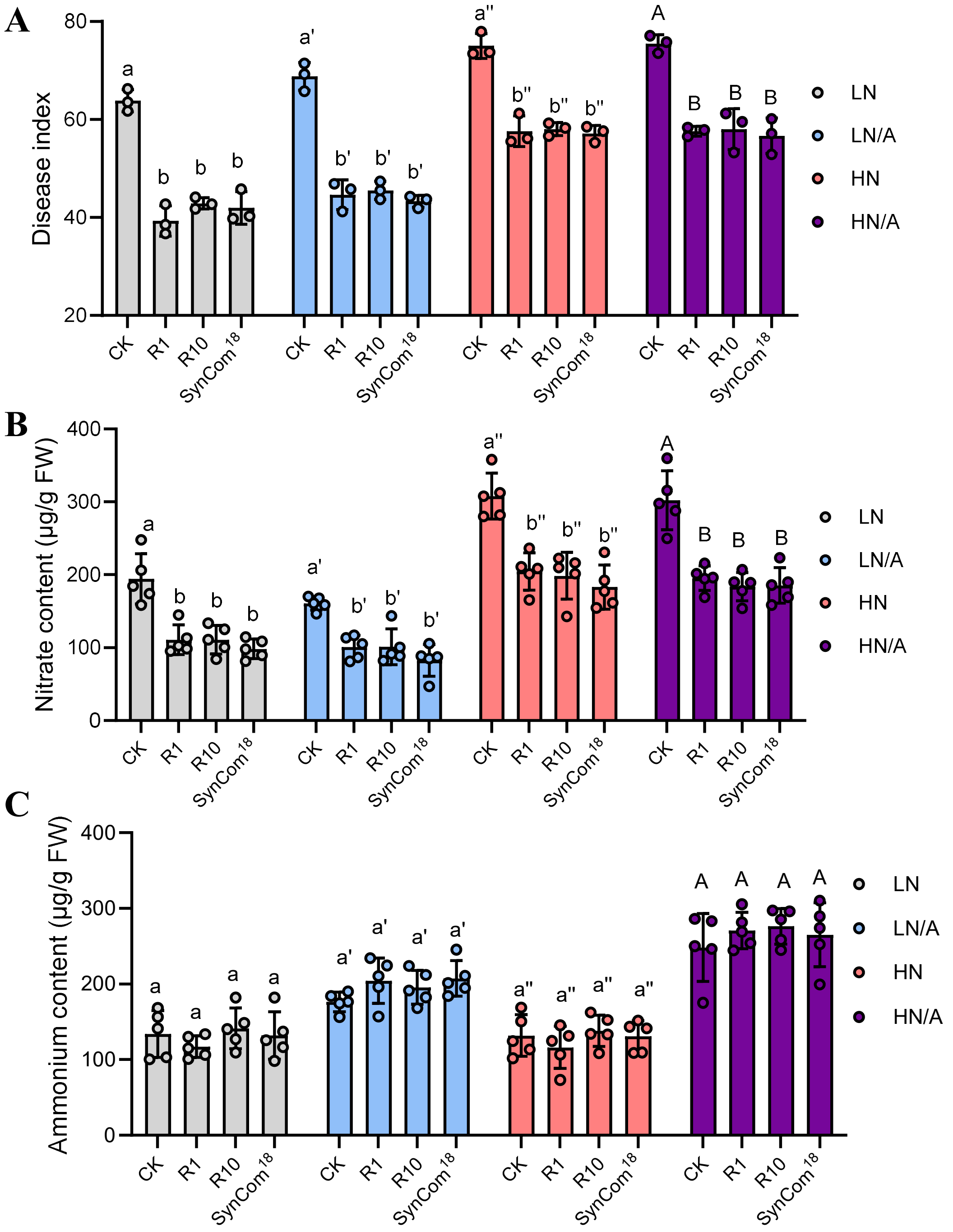

Figure S9. Denitrifying bacteria reduce nitrate content in the rhizosphere soil.

Rapeseed plants were treated with four different nitrogen regimes: LN: 6 mM NO_3^-; HN: 30 mM NO_3^-–N; LN/A: 6 mM total N (NH_4^+–N:NO_3^-–N = 7:3); HN/A: 30 mM total N (NH_4^+–N:NO_3^-–N = 7:3). Simultaneously, denitrifying bacteria R1, R10, and SynCom18 were inoculated. After 28 days of treatment, the disease index (A), nitrate content (B), and ammonium content (C) in the rhizosphere soil were measured. Disease index evaluation: n = 32 biological replicates (randomized into 3 analytical groups); soil nitrogen measurements: n = 5 replicates per condition. Data in A, B, and C are presented as mean ± SD. Statistical significance was determined by one-way ANOVA with Duncan's multiple range test (different letters indicate significant differences at $P < 0.05$).

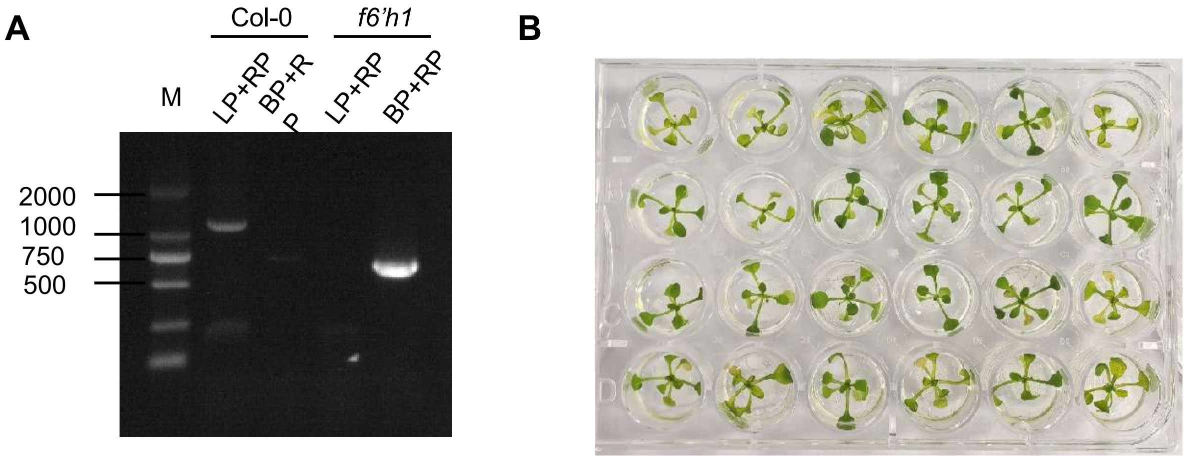

Figure S10. Identification and growth characteristics of the *Arabidopsis* mutant *f6'h1*. (A) The identification of *f6'h1* using the tri-primer method. (B) The growth status of *f6'h1* in hydroponic culture in a 24-well plate after 14 days.

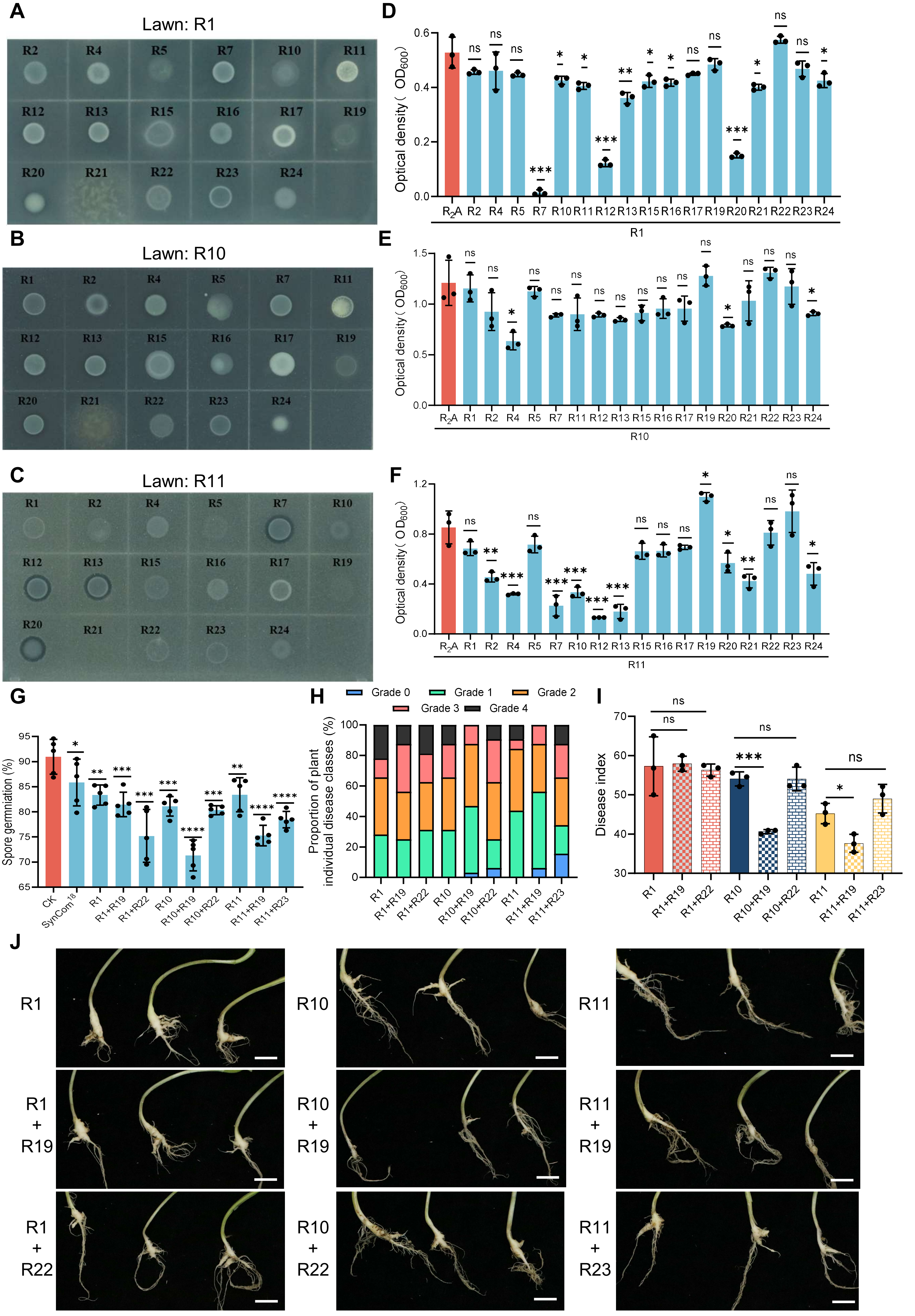

A
Lawn: R1
D
Optical density (OD_{600})
R1
B
Lawn: R10
E
R10
C
Lawn: R11
F
R11
G
Spore germination (%)
H
Grade 0 Grade 1 Grade 2 Grade 3 Grade 4
Proportion of plant individual disease classes (%)
I
Disease index
J
R1
R10
R11
R1 + R19
R10 + R19
R11 + R19
R1 + R22
R10 + R22
R11 + R23

Figure S11. Evaluation of the effects of dual bacterial combinations on the biocontrol of clubroot disease. (A-F) Binary inter-bacterial inhibition. Inhibition assays were conducted on R2A plates, showing the inhibitory effects of R1 (A), R10 (B), and R11 (C) on bacterial species identified in SynCom[18]. Photographs were taken after 2 days of co-culture. (D-F) OD_{600} values of SynCom[18] strains co-cultured for 24 hours with fermentation broths of R1 (D), R10 (E), and R11 (F). (G) Germination rates of resting spores after 3 days of treatment with fermentation broths of single strains, dual-strain combinations, or SynCom[18]. (H-J) Pot experiment evaluating the biocontrol efficacy of single strains or dual-strain combinations against clubroot disease. Disease severity grading (H), disease index (I), and root phenotypes were assessed 20 days after bacterial treatment. The number of biological replicates: Panel B, D, F, and I: n = 3, Panel G: n = 5, Panel H: n = 32. Bars in panel J are 2 cm. Data in panels D, E, F, G, and I are presented as mean ± SD. Statistical significance was determined using a two-tailed Student's *t* -test: $^{*}P < 0.05$, $^{**}P < 0.01$, $^{***}P < 0.001$.

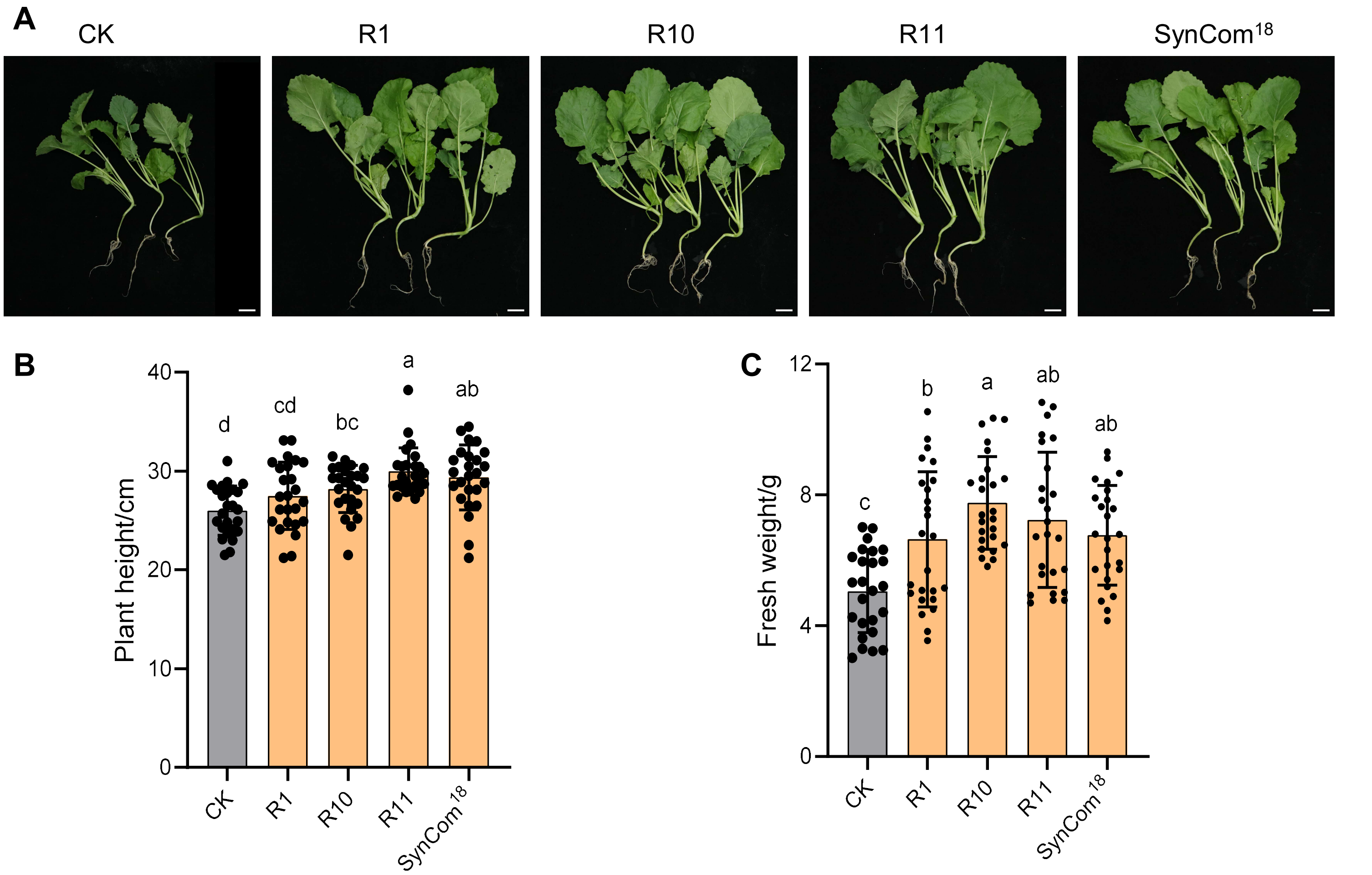

Figure S12. Denitrifying bacteria promote the growth of rapeseed.

After 28 days of treatment with single bacteria and SynCom[18], the growth phenotype (A), plant height (B), and fresh weight (C) of rapeseed are shown. Bar = 3 cm, n=25. Data in B and C are presented as mean ± SD. Statistical significance was determined by one-way ANOVA with Duncan's multiple range test (different letters indicate significant differences at $P < 0.05$).
